# Impact of Temperature Regimes and Smart Packaging on Volatilome Evolution During the Shelf Life of *Agaricus bisporus*

**DOI:** 10.3390/jof12070477

**Published:** 2026-06-30

**Authors:** Mehdi Moayedi, Michele Pedrotti, Iuliia Khomenko, Emanuela Betta, Andrea Romano, Matteo Tonezzer, Luca Cappellin, Franco Biasioli

**Affiliations:** 1 Research and Innovation Centre, Fondazione Edmund Mach, Via Edmund Mach 1, San Michele all’Adige, 38010 Trento, Italy; mehdi.moayedi@studenti.unipd.it (M.M.); iuliia.khomenko@fmach.it (I.K.); emanuela.betta@fmach.it (E.B.); andrea.romano@fmach.it (A.R.); matteo.tonezzer@fmach.it (M.T.); luca.cappellin@unipd.it (L.C.); franco.biasioli@fmach.it (F.B.); 2 Department of Chemical Sciences, University of Padua, Via Marzolo 1, 35131 Padova, Italy

**Keywords:** *Agaricus bisporus*, shelf life, volatile organic compounds (VOCs), proton transfer reaction-mass spectrometry (PTR-MS), smart packaging

## Abstract

*Agaricus bisporus* is highly valued for its sensory and nutritional aspects, but it is highly perishable because of its intense postharvest metabolism. In this study, we looked at how white button mushrooms responded to three thermal regimes including constant refrigeration (4 °C), ambient storage (20 °C), and repeated temperature stress (RTS). The investigations were combined with an innovative smart packaging called Store Box (SB). Using an integrated volatilomics approach combining both gas chromatography–mass spectrometry (GC-MS) and proton transfer reaction time of flight mass spectrometry (PTR-ToF-MS), we tracked volatile compounds from intact mushrooms and internal tissues and linked these with weight loss and CO_2_ production. According to the results, the SB stabilizes the internal microenvironment by reducing moisture loss and modulating gas exchange through controlled CO_2_ buildup. This mechanism attenuated the respiratory surge and significantly delayed the rise of spoilage-related markers like methanethiol, acetaldehyde, and hexanal. At the same time, SB preserved freshness indicators such as 1-octen-3-one and benzaldehyde, which normally fade as mushrooms age. The protective effect was especially clear during thermal fluctuations, where SB acted as a metabolic buffer. Overall, this work offers new insights into the volatilome dynamics of *Agaricus bisporus* and confirms that smart packaging can help offset the damage caused by temperature instability along the supply chain.

## 1. Introduction

*Agaricus bisporus* (white button mushroom) is the most widely cultivated edible mushroom worldwide, accounting for a substantial share of global production and consumption [1]. Its commercial relevance is driven by a favorable nutritional profile, including high protein content (~29% dry matter, DM), low fat levels, and the presence of essential micronutrients, as well as its characteristic aroma and desirable texture [1,2]. Despite this economic importance, *A. bisporus* is highly perishable, and its shelf life is limited to a few days at ambient temperature [1,3].

This limited shelf life is closely linked to mushroom physiology. Unlike most horticultural products, *A. bisporus* lacks a protective cuticle, leaving tissues directly exposed to environmental exchange [4,5]. Coupled with a high-water content (85–95%) and elevated respiration rates, this structural feature results in rapid moisture loss, tissue softening, enzymatic browning driven by polyphenol oxidase activity, and susceptibility to microbial spoilage [1,5,6]. Consequently, postharvest quality is governed by tightly coupled physiological processes, including water loss, respiratory metabolism, and biochemical degradation [4].

Refrigeration (0–4 °C) is the primary strategy to slow these processes, extending shelf life to approximately 5–7 days [1]. However, low-temperature storage introduces trade-offs, including chilling-induced damage, membrane destabilization, and surface browning [4]. Moreover, real-world cold chains are frequently disrupted by temperature fluctuations during transport and retail [7]. Such RTS events are not merely experimental constructs; rather, they represent a widespread and economically relevant challenge. These fluctuations induce transient metabolic surges, promote condensation within packaging systems [8], and accelerate both microbial proliferation and physiological deterioration [4,9]. Therefore, understanding the metabolic response of *Agaricus bisporus* to RTS is essential for designing robust postharvest strategies.

At the packaging level, modified atmosphere packaging (MAP) is widely employed to modulate O_2_ and CO_2_ concentrations and slow senescence [10,11]. For *Agaricus bisporus*, atmospheres with reduced O_2_ (<10%) and controlled CO_2_ (≤5%) are generally considered optimal. Under such conditions, shelf life can be extended to 8–14 days. However, the effectiveness of MAP strongly depends on the balance between gas permeability and respiration rate [10,12,13]. Deviations from this balance, such as excessive CO_2_ accumulation or severe O_2_ depletion, can induce anaerobic metabolism, off-flavor formation, and tissue damage [11,13]. More recently, smart packaging systems have been developed to stabilize the internal microenvironment through passive atmosphere modulation, enabling real-time monitoring of temperature, relative humidity, and gas composition. These systems represent a promising approach for mitigating environmental fluctuation. However, their effectiveness must be evaluated by assessing the specific metabolic responses they induce.

In this context, volatilomics offers a powerful framework for investigating postharvest processes. Volatile organic compounds (VOCs) are not only key contributors to mushroom aroma but also sensitive indicators of metabolic activity and physiological state [14]. The characteristic aroma of *Agaricus bisporus* is dominated by C8 compounds, such as 1-octen-3-ol, 3-octanone, and 1-octen-3-one, which are generated from linoleic acid through the lipoxygenase/hydroperoxide lyase pathway. Other contributors include aromatic and aldehydic compounds such as benzaldehyde and benzyl alcohol [15,16,17,18]. Importantly, the volatilome evolves dynamically during storage, reflecting a shift from fresh, lipid-derived metabolites toward compounds associated with fermentation, oxidation-derived and microbial activities including ethanol, acetaldehyde, and methanethiol [4,9,11,14].

Conventional GC–MS enables detailed identification of VOCs but is generally limited to static and destructive measurements, providing only a snapshot of the system [19]. In contrast, PTR-MS enables rapid, sensitive, and non-invasive monitoring of VOC emissions, allowing real-time tracking of metabolic processes in intact samples [20,21]. This approach has been successfully applied to characterize species-specific and degradation-related volatile profiles in fungal material [22,23]. Combining these two techniques offers the opportunity to move beyond descriptive profiling toward a more mechanistic understanding of volatilome dynamics.

In the present study, we apply an integrated volatilomics strategy that combines dynamic headspace PTR-ToF-MS measurements on intact mushrooms with static headspace analysis (PTR-ToF-MS and GC–MS) on disrupted tissues. These complementary approaches provide distinct but interconnected information: dynamic measurements capture real-time emissions reflecting the physiological state of intact samples, while static analyses resolve internal biochemical transformations associated with tissue degradation. By integrating volatilome data with physiological parameters (CO_2_ production, weight loss, and dry matter), we aim to characterize the metabolic trajectories of *A. bisporus* under different thermal regimes (4 °C, 20 °C, and repeated temperature stress) and to determine how smart packaging modulates these processes. This framework provides a mechanistic basis for understanding postharvest deterioration and supports the development of more effective packaging strategies for mushroom supply chains.

## 2. Materials and Methods

### 2.1. Materials

#### 2.1.1. Samples

Fresh *Agaricus bisporus* samples (46 kg) were purchased from Funghi Valbrenta s.r.l. (Valbrenta, Italy). Mushrooms were harvested and cleaned of the substrate debris. Only mature, healthy, and undamaged button mushrooms with caps (pilei) uniform in size were selected. The mushrooms were packed in 500 g polypropylene plastic trays sealed with Omni PW A469 stretch film (Omni Group, Keilor Park, Australia and placed in crates, each holding approximately 4 kg. Half of the crates were placed into SB, while the other half remained in the original plastic trays (STD). All mushrooms were transported together to the laboratories of Fondazione Edmund Mach (San Michele all’Adige, Italy).

#### 2.1.2. The Store Box, a Reusable Packaging System

The SB container is a smart, stackable and reusable packaging system designed within the SISTERS EU project. It aims to enhance the storage and transportation conditions of packed food products across different stages of the food supply chain. In practice, the SB is a polypropylene container with external and internal dimensions 60 × 40 × 32 cm and 57 × 37 × 30 cm, respectively. As secondary packaging it is designed to maintain stable internal ambient conditions and extend shelf life through a passive modified atmosphere. It is integrated with sensor technologies (Rebus Labs, Stettlen, Switzerland) to offer continuous on-line and non-invasive environmental monitoring (temperature, humidity, O_2_, CO_2_) and to support data-driven logistics for fresh and highly perishable food products. The SB modularity ensures that individual units can be replaced or upgraded without disrupting the wider network. This concept is central to the scalability of the solution. Each SB is assigned a Unique Digital Identifier (UID). This identifier allows the unit to maintain a persistent digital history (provenance) and interact seamlessly with different software platforms. Furthermore, it enables plug-and-produce functionality within decentralized logistics or data networks. This system was selected for the study to evaluate a circular logistics model designed to reduce packaging waste, while simultaneously allowing for the correlation of real-time environmental fluctuations with spoilage dynamics.

To monitor the environmental conditions of the samples stored in STD containers, and to validate the data collected by the SB sensor kits, additional reference instruments were used. Temperature, relative humidity, and CO_2_ were recorded using OM-92 and OM-CP-RFCO2RHTEMP2000A devices (Omega Engineering Limited, Manchester, UK), while O_2_ concentrations were measured with a Dräger Pac 6500 monitor (Drägerwerk AG & Co. KGaA, Lübeck, Germany).

### 2.2. Methods

Upon arrival, the mushrooms were assigned to three different storage conditions, namely room temperature (about 20 °C), refrigerated temperature (4 °C, 90–95% relative humidity, RH) and RTS conditions. The mushrooms assigned to the RTS condition were taken out of refrigerated storage every 2 days for 6 h. They were then reintroduced into cold storage to simulate cold chain interruptions. Sampling for instrumental analysis was performed on days 1, 5, 8, and 15. Due to the rapid deterioration of the samples at ambient temperature, this condition was monitored for only one week, whereas the other storage conditions were monitored for 15 days. Measurements were divided into two categories. First, non-destructive monitoring of intact samples to assess weight loss (WL), CO_2_ production, and real-time VOC analysis. Second, destructive analyses were conducted on disrupted tissues to determine dry matter (DM), and internal metabolite profiling.

#### 2.2.1. Nondestructive Measurement

##### Weight Loss

To measure WL, at the beginning of the experiment three packs were taken from each of the four batches, marked, and weighed. At every sampling, the weight of each assigned pack was noted. The difference between the weight on arrival day and the weight on the sampling day was calculated employing the following formula.

(1) $$WL\;\left[\%\right]=\frac{W_{0}-W_{t}}{W_{0}}\times 100$$

where $W_{0}$ is the weight of the tray at the arrival, and $W_{t}$ is the weight at the sampling day.

##### VOCs Sampling

For each sampling, nine mushrooms were randomly selected and divided into three groups as biological replicates. Each group was placed in a 250 mL glass container and kept at room temperature for 2 h for acclimation. Then, each jar was sealed for 30 min for headspace incubation before VOC sampling. Two different measurements were performed. First, VOC emission was monitored using (PTR-ToF-MS 8000, Ionicon, Innsbruck, Austria). PTR-ToF-MS enables real-time, non-destructive monitoring by soft proton-transfer ionization, followed by time-of-flight mass separation of the generated product ions. Second, CO_2_ emission was measured as an indicator of mushroom respiration using a (LI-850, LI-COR, Bad Homburg, Germany) gas analyzer. VOCs emission was measured for 1 min after the incubation, while for CO_2_ production, each sample was measured for 10 s immediately after the closure of the glass container and for 30 s immediately after VOCs measurement.

The respiration rate of *Agaricus bisporus* during storage was monitored through CO_2_ (µmol/mol) emission, measured in the dynamic headspace using a Li-850 CO_2_/H_2_O gas analyzer connected to a PC with the (Gas Analyzer software (1.0.2), Lincoln, NE, USA). The measurement was conducted immediately after PTR-ToF-MS analysis by using the same jars (without opening them) to maintain consistency in the sampling conditions. Sampling was performed through a 1/16-inch Peek inlet.

For non-destructive VOC analysis by PTR-ToF-MS, H_3_O^+^ was used as the primary ion. The headspace of each sealed jar was directly sampled through the PEEK inlet connected to the PTR-ToF-MS 8000 instrument. Measurements were performed under standard PTR-ToF-MS operating conditions, with a reduced electric field (E/N) of 128 Td and a heated inlet system to prevent condensation effects, as described in previous studies [24]. VOC emissions were recorded 60 s per sample, providing a rapid fingerprint of the volatile profile emitted by intact mushrooms.

#### 2.2.2. Destructive Measurements

##### Dry Matter

DM content was determined at each sampling day by selecting four mushrooms per batch. Samples were weighed, oven-dried at 105 °C for 48 h (until constant weight), and reweighed. DM was expressed as the percentage of fresh weight (Formula (2)).

(2) $$DM\;[\%]=\frac{W_{t}}{W_{0}}$$

where $W_{0}$ is the weight before the drying and $W_{t}$ is the weight after drying.

##### VOCs Sampling

For the headspace analysis, at each sampling day, a sample of 100 g of mushrooms was taken from each treatment and frozen in liquid N_2_ and thus homogenized into a fine powder using a laboratory mill (A11 n IKA Mill, Staufen, Germany). In total, 1 g of the obtained powder was then transferred to a 20 mL GC vial that contained 1 mL antioxidant solution composed of 0.25 g citric acid, 0.25 g ascorbic acid, 20 g NaCl, and 50 g water. Consequently, the prepared vials were kept at −80 °C until analysis.

Static headspace measurements were performed using both PTR-MS and GC–MS. For PTR-MS analysis, samples were analyzed in triplicate using the same PTR-ToF-MS 8000 instrument and operational conditions described above and previously reported in detail [25].

For GC–MS analysis, after defrosting the samples and adding 0.05 mL of internal standard (2-octanol, 5 mg/L), the vials were put in a cooler (10 °C) thermostatic autosampler. The equilibration time was 10 min at 40 °C. The SPME extraction time was 30 min. The fiber used was a 1 cm PDMS/DVB. The analytes were desorbed from the SPME fiber at 240 °C in the injector port of the GC (Agilent 7890B) and separated on a DB-ax UI (30 m × 0.25 mm × 0.25 μm, Agilent, Folsom, CA, USA) column. The GC oven temperature program (44 min) began at 40 °C for 1 min, then increased to 220 °C at 5 °C per minute, then increased to 240 °C at 10 °C per minute and finally held at 240 °C for 5 min. Helium was used as the carrier gas with a constant column flow rate of 1.5 mL per minute. The GC was interfaced with a mass detector, which operated in electron ionization mode (EI, internal ionization source; 70 eV) with a scan range from *m*/*z* 35 to 350 (Agilent MS 5977A MSD). The volatile compounds were identified by comparing their mass spectra with data from libraries (NIST Standard Reference Database 1A, 2014) using linear retention indexes (LRI) as a reference.

### 2.3. Data Processing and Statistical Analysis

To validate sensors’ performance, the Root Mean Square Error (RMSE) for each of the storage conditions was calculated. PTR-ToF-MS data processing, calibration, and quantification were carried out according to known protocols that were previously thoroughly explained by [21,25]. In summary, VOC concentrations were determined under the assumption of a constant reaction rate coefficient (k_R_ = 2 × 10^−9^ cm^3^ s^−1^) using dead time correction, internal mass calibration, and peak extraction [21]. After data calibration, a mass extraction procedure was applied, and 288 mass peaks were extracted from the PTR-ToF-MS non-destructive and 280 from the destructive PTR-ToF-MS datasets, respectively. Afterwards, univariate comparison between the BLANK and the other two sample types (STD and SB) was performed employing Student’s *t*-tests to identify mass peaks that were significantly different than the BLANK samples (*p*-value < 0.05), combined with visual inspection. The significant different mass peaks were employed for further statistical analysis.

For both PTR-MS datasets, a principal components analysis (PCA) was performed after applying a logarithmic transformation, log (x + 1), followed by mean centering of the data. In addition, univariate analysis was carried out by performing one-way ANOVA to evaluate significant differences among treatments. This was followed by Tukey’s Honest Significant Difference (HSD) test (*p*-value < 0.05) for pairwise comparisons for each *m*/*z* and compound in the PTR-ToF-MS and GC–MS datasets. Because signal intensities spanned a wide dynamic range, statistical tests were performed on log_10_-transformed values and the interquartile range (IQR) based outlier rule was applied. Based on the IQR method, values falling below Q_1_ − 1.5 × IQR or above Q_3_ + 1.5 × IQR were excluded.

Because of the high dimensionality of the volatilome data and the large number of detected features, the most biologically significant *m*/*z* peaks and volatile compounds (10–12 markers per dataset) were selected for presentation in the main text. These markers were identified based on their contribution to the variance in the PCA and their statistical significance in the one-way ANOVA. The comprehensive datasets, containing the full profile from the destructive analysis (GC–MS) across all treatments and all mass peaks significantly different from the blank sample in the dynamic and static headspace (PTR-ToF-MS), are provided in the Supplementary Materials (Supplementary Tables S1–S9).

All statistical analyses were conducted with R (v4.5.1; R Core Team, 2024) setting a significance level of α = 0.05. Data preprocessing and structuring were carried out using the tidyverse and openxlsx packages. Visualization was performed with ggplot2, and multivariate analyses were conducted with factoextra. One way ANOVA analyzed with Tukey’s post hoc comparisons and estimated marginal means were obtained with emmeans and compact letter displays were generated with multcompView.

## 3. Results and Discussion

To resolve the complex dynamics of postharvest deterioration, we integrated non-destructive real-time monitoring with destructive tissue analysis. By distinguishing between the external volatilome of intact mushrooms and the internal biochemical markers of damaged tissues, we were able to characterize both the surface physiological signals and the deep-seated metabolic transformations governing the shelf life of *Agaricus bisporus*.

### 3.1. Non-Destructive Physiological Monitoring

The internal microenvironment across all storage regimes was modified effectively by the SB. Under refrigerated storage, temperatures stabilized around 4 °C within 10–20 h. Inside the SB, temperatures were slightly higher than in the cold room (ΔT ≈ 1 °C), with small fluctuations corresponding to box openings for sampling. The SB maintained near-saturated humidity (≈100%) after a brief initial rise, whereas RH in the STD condition ranged between 80–95%, reflecting lower moisture retention. Both CO_2_ and O_2_ inside the SB remained at baseline from ambient cold-room conditions (CO_2_ ≈ 0.04%; O_2_ ≈ 20.9%). Under RTS conditions, temperature spikes (>15 °C) occurred during ambient exposure, returning to ~4 °C between cycles. The STD samples showed slightly higher concentration and greater variability for CO_2_ and O_2_, consistent with a more open surrounding environment. The validation metrics demonstrate strong agreement between the SB sensor and the reference instruments. The RMSE values for temperature and relative humidity were 0.7 °C and 2.5% for the refrigerated condition, 0.7 °C and 4.1% for ambient storage and 1.6 °C and 3.8% for the RTS condition. Representative photographs of *Agaricus bisporus* stored in SB and STD packaging under ambient, refrigerated, and RTS conditions are provided in Supplementary Figures S1 and S2 to visually document the physical appearance of the samples before and during storage. Compared to STD, the SB demonstrated superior humidity recovery, returning to ≈100% RH after each stress event compared to 70–90% in STD samples. In SB, and under RTS condition, the gas composition shifted and stabilized at ~2% CO_2_ with a 1–2% O_2_ depletion after two days during exposure to ambient conditions. At room temperature (20 °C), thermal profiles were identical for all packaging types, rising to ~20 °C within 24 h and remaining stable thereafter; no differences were detected between SB and STD conditions during the one-week shelf life. In contrast, humidity levels varied significantly. The SB samples retained 95–100% RH. In contrast, the humidity levels in the STD samples dropped to about 70%. In SB samples, O_2_ declined to about 17.5%, and CO_2_ accumulated significantly (>7%) despite periodic openings for sampling. This accumulation is consistent with elevated respiration rates and the gas-barrier properties of the SB system, which limited CO_2_ dissipation into the surrounding environment.

**Figure 1:**
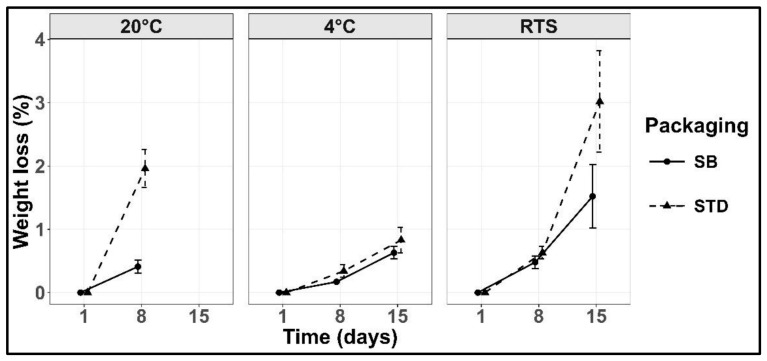
WL percentage for the mushroom stored in different conditions.

Across all storage conditions, WL increased progressively throughout the shelf life (Figure 1). By Day 8, the highest weight loss was observed in STD samples stored at 20 °C. This outcome aligns with the higher metabolic activity and elevated transpiration rates typically triggered by ambient temperature storage. Notably, ambient conditions lead to accelerated moisture migration from the mushroom tissue. Compared to STD, SB samples exhibited significantly lower WL percentages across all temperature regimes. For instance, in samples stored at 20 °C, the SB packaging reduced weight loss fourfold compared to STD packaging, while under RTS conditions it halved the WL compared to the STD samples. These results indicate that SB packaging limits moisture loss and stabilizes the internal microenvironment, even under thermal stress. This performance is consistent with the internal humidity data. This result is consistent with the results presented in previous studies showing that humidity control is a critical factor in fresh mushroom packaging. Mahajan et al. reported that excessive in-package moisture can promote condensation, microbial growth, and discoloration, whereas insufficient humidity accelerates weight loss and undesirable textural changes [8]. In this context, the SB system appears to provide a balanced moisture-retention effect, limiting desiccation and weight loss while maintaining a stable high-humidity microenvironment.

**Figure 2:**
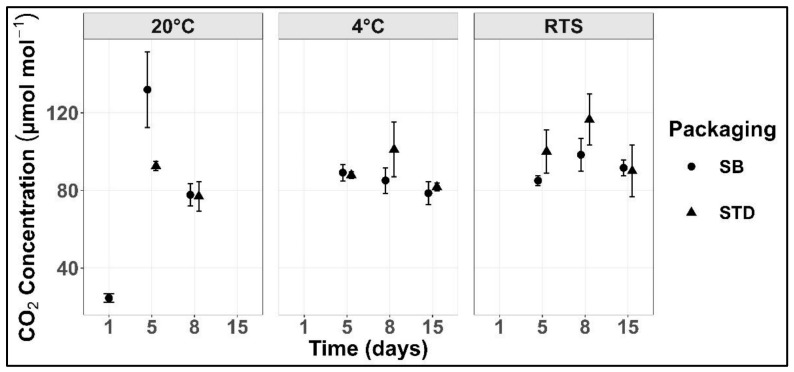
CO_2_ production of mushrooms over 15 days across storage temperatures and packaging types. STD and SB represent Standard and Store Box packaging.

Figure 2 shows CO_2_ production of mushrooms across different storage temperatures and packaging types during the storage period. At the beginning of the shelf life (Day 1), all samples consistently showed low CO_2_ concentrations between 20 and 28 µmol mol^−1^, representing a physiological baseline across all treatments. During the storage period, CO_2_ concentrations varied markedly based on packaging type and storage conditions.

On Day 5, a substantial increase in CO_2_ was observed under ambient conditions. Notably, samples in SB reached higher CO_2_ levels than those in STD. This does not reflect greater metabolic activity. Instead, it is a consequence of the gas-barrier properties of the SB system, which limits CO_2_ dissipation into the surrounding environment. This retained CO_2_, combined with reduced O_2_ availability, creates a buffered internal atmosphere that in turn attenuates oxidative metabolism. After Day 5, CO_2_ concentrations declined in both packaging types, likely due to surface dehydration, tissue senescence, and a general decline in respiratory activity at later stages.

In refrigerated storage (4 °C), both SB and STD samples exhibited low and stable CO_2_ production throughout the shelf life, consistent with suppressed respiration at low temperatures [1]. However, SB packaging consistently maintained slightly lower CO_2_ levels, attributable to improved internal humidity buffering and more effective microenvironmental modulation, which helps stabilize metabolic activity and delay senescence [8,10].

Under RTS conditions, CO_2_ production exhibited a bell-shaped temporal pattern with a delayed peak compared to constant ambient storage. This delay is attributed to the lower baseline temperature, which initially suppressed respiration before warming phases triggered transient metabolic surges [1]. By Day 8, CO_2_ levels in STD samples increased sharply, with wider dispersion between replicates suggesting a loss of metabolic control. In contrast, SB packaging displayed a more moderate and uniform trend, indicating that its physical structure functioned as a thermal and gaseous buffer, partially shielding the fungi from warming-induced metabolic accelerations. After Day 8, CO_2_ levels declined in both packaging types, potentially due to substrate depletion, tissue senescence, or a metabolic shift toward partial anaerobic responses [4,11]. Overall, while SB packaging does not entirely prevent RTS-induced spoilage, it effectively slows respiration-driven deterioration. CO_2_ production trends were consistent with weight loss data, reinforcing the interpretation that SB packaging modulates both gas exchange and moisture loss through a common microenvironmental buffering mechanism [8]. Adjusting packaging permeability through micro-perforation can influence both the O_2_/CO_2_ balance and the formation of off-flavor volatiles in button mushrooms [11]. Therefore, the moderated CO_2_ accumulation observed in SB samples may reflect a comparable regulation of respiratory metabolism, complementing its moisture-retention effect. Conventional MAP studies have mainly evaluated shelf-life extension through endpoint quality attributes such as weight loss, color, veil opening, and texture [10]. Whereas active packaging systems have focused on engineering film permeability to maintain target O_2_/CO_2_ conditions [26]. The SB system was assessed by combining these physiological indicators with real-time VOC monitoring.

**Figure 3:**
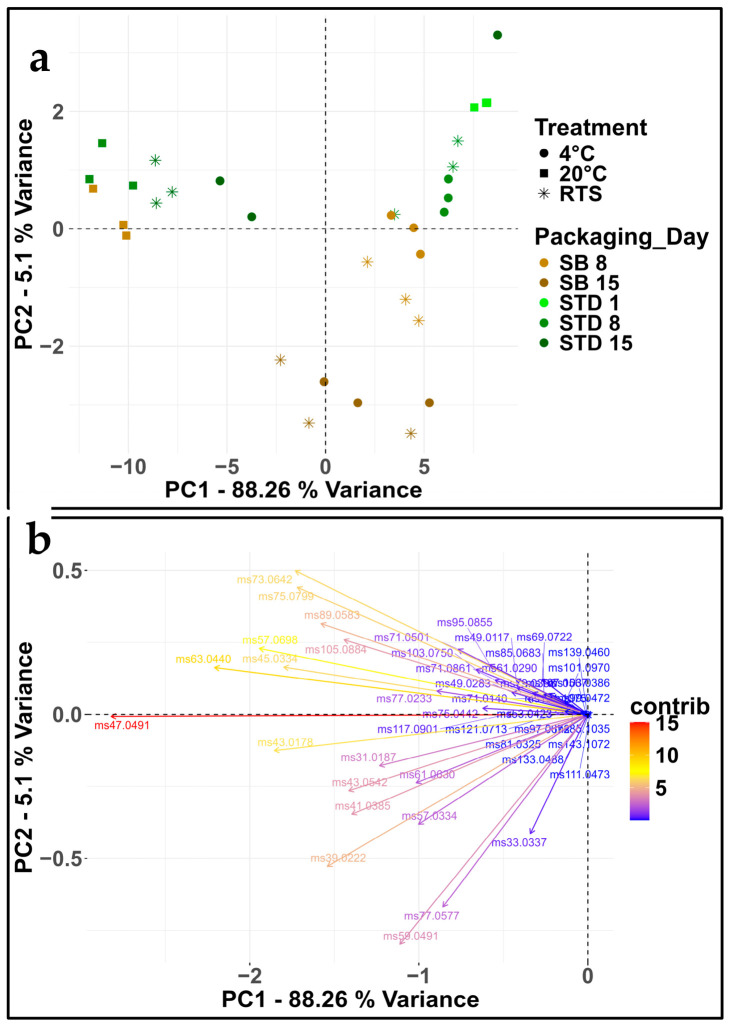
(**a**) Principal Component Analysis (PCA) score plot of the nondestructive PTR-ToF-MS measurement, illustrating VOC emission trends of intact mushrooms across different packaging and storage conditions. (**b**) Corresponding loading plot highlighting the primary VOCs driving the separation along PC1 and PC2.

To investigate the underlying biochemical and metabolic changes driving the physiological trends described above, the temporal evolution of VOCs was monitored non-destructively by PTR-ToF-MS. This approach allows real-time tracking of volatile emissions from intact mushrooms, providing a direct window into the metabolic shifts associated with senescence and packaging effects. The PCA score plot (Figure 3a) illustrates the time trends of VOC emission in the intact samples, with the STD samples measured at the initial time point (Day 1) serving as the baseline. PC1 and PC2 explain 88.26% and 5.1% of the total variance, respectively. A shift toward negative PC1 values (leftward) reveals spoilage progress in time, and PC2 reveals minor additional variance based on less dominant metabolic changes or subtle treatment effects. For the STD samples at both Day 8 and Day 15 (under ambient and RTS conditions), a marked leftward shift in the PCA score plot was observed, indicating higher VOC production resulting from respiration and degradation processes during storage. Notably, by Day 8 both STD and SB samples stored at 20 °C had already migrated to the far left of the score plot, suggesting that at ambient temperature the rapid metabolic acceleration leads to a complete transformation of the volatile profile within the first week, exceeding the buffering capacity of both packaging systems.

In contrast, SB samples showed a limited shift in the VOCs implying that SB packaging effectively suppresses spoilage-related VOC release. Under RTS conditions, STD samples showed a large leftward shift comparable to ambient storage, while SB samples displayed a smaller and predominantly downward shift along PC2, remaining closer to the Day 1 reference points. This divergence confirms that SB packaging partially preserves the original volatile fingerprint even under repeated thermal stress. Compared to the other storage temperatures, the refrigerated samples (4 °C) clustered closer to the origin and slightly to the left, particularly on Day 8, indicating limited volatilome variation because of reduced metabolic activity under cold storage. The loading plot of the nondestructive PTR-MS measurements (Figure 3b) highlights a small subset of VOCs as the major separation contributors along PC1 and PC2. The color scale (“contrib”) reflects the relative contribution of each variable to PC1 and PC2, calculated from the squared loadings. Tentatively identified (t.i.) ethanol (*m*/*z* 47.049) is the most prominent contributor along PC1. As a well-established marker of spoilage and fermentative respiration, its prominence confirms the onset of spoilage and drives the variation across storage time and packaging conditions [11]. Another key contributor in PC1 is *m*/*z* 45.033 (acetaldehyde), which is a direct metabolic precursor to ethanol [11,14]. Additional mass peaks at *m*/*z* 63.044 and *m*/*z* 75.079 belonging to water clusters of acetaldehyde and ethanol respectively were also detected and contributed to PC1.

**Figure 4:**
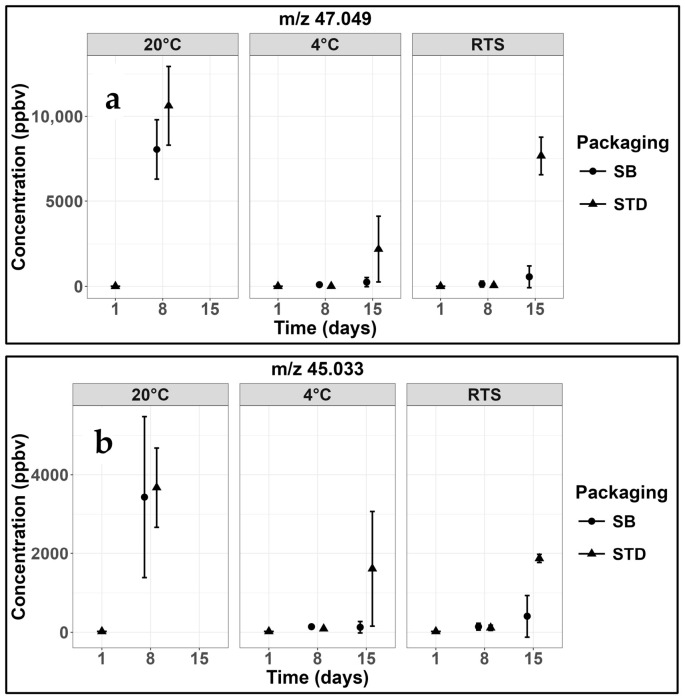
Concentrations of (**a**) ethanol and (**b**) acetaldehyde in 3 different conditions, and the two packaging in nondestructive PTR-ToF-MS measurement.

Figure 4 shows the concentrations of ethanol (*m*/*z* 47.049) and acetaldehyde (*m*/*z* 45.033) nondestructively measured by PTR-ToF-MS measurement. The concentration of ethanol and acetaldehyde shows an increasing trend over time in all three conditions in both packaging types (STD and SB). The concentration of both compounds increases moderately in refrigerated conditions compared to the RTS condition after 15 days. On the other hand, the ambient temperature led to a much greater increase in the concentration of the two compounds. At ambient temperature, the concentrations of ethanol and acetaldehyde reached more than 10,000 ppbv and approximately 3500 ppbv in STD packaging after only eight days of storage. In SB packaging, the concentrations of ethanol and acetaldehyde reached around 7500 ppbv and almost 2000 ppbv, respectively, showing a smaller increase compared to the STD mushrooms. Under refrigerated conditions, both compounds remained low through Day 8. However, by Day 15 a clear divergence emerged between packaging types. The STD samples showed a significant increase in both ethanol and acetaldehyde, while SB samples maintained consistently low concentrations, confirming the protective effect of the smart packaging even during extended cold storage. Under RTS conditions, the increase in ethanol in STD samples on Day 15 was even more pronounced than under refrigeration, reflecting the cumulative metabolic impact of repeated thermal fluctuations.

### 3.2. Destructive Analysis: Dry Matter Content and Volatile Profiling

**Figure 5:**
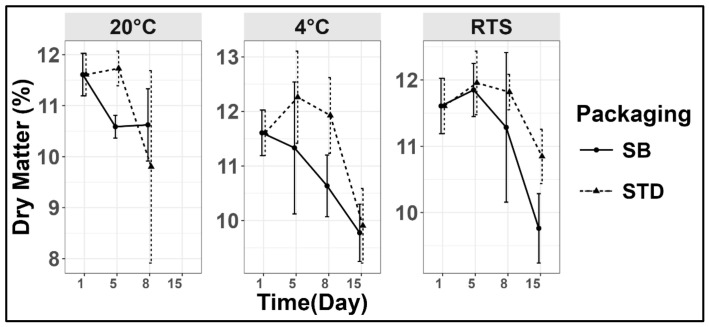
DM for the mushroom stored in different conditions (20 °C, 4 °C and Repeated Temperature Stress) and in different containers (SB and STD).

Following the non-destructive VOC analysis, destructive measurements were conducted to quantify the internal physiological changes associated with senescence. The DM content of the mushroom samples was measured in the different storage conditions over time, and the results were presented in Figure 5.

Initially, DM content was approximately 12%. In the SB samples, this value gradually decreased over the shelf life. The decline was driven by the rapid respiratory consumption of non-structural carbohydrates such as mannitol (a polyol), followed by glycogen (a polysaccharide) and trehalose (a disaccharide) [1]. The depletion of these reserves reduces cellular turgor and hyphal density, resulting in WL and texture deterioration [12]. This biochemical depletion therefore contributes to weight loss independently of desiccation and represents an intrinsic metabolic cost of storage that packaging alone cannot prevent.

In contrast, the STD samples exhibited an initial increase in DM up to Day 5. This rise is attributed to rapid water loss (Figure 1), which concentrates the internal solids faster than respiration can deplete them. For the mushrooms stored at ambient temperature (20 °C), this trend reversed sharply after Day 5, leading to a pronounced drop in DM by the end of the storage period (Day 8). In other cases, the higher water loss in STD conditions maintained a higher DM percentage compared to the SB group throughout the shelf life. This is especially visible for the samples stored under RTS conditions, which ended the experiment with significantly higher DM than the other mushrooms.

To complement the non-destructive VOC monitoring and resolve compound-specific changes at the tissue level, the volatile profile was further investigated by destructive GC–MS analysis. The overall trends were consistent with previous analyses: samples stored at 20 °C exhibited the fastest and most extensive spoilage, while refrigerated samples (4 °C) showed significantly slower VOC evolution due to suppressed respiration and enzymatic activity. Table 1 presents the one-way ANOVA comparison of key VOC markers in mushrooms stored under RTS conditions in both STD and SB packaging across the four time points (Days 1, 5, 8, and 15). Values represent mean ± standard deviation (ppbv). The associated Tukey grouping letters indicate statistically significant differences (*p* < 0.05) across the shelf life.

At the beginning of storage (Day 1), typical mushroom markers such as 1-octen-3-ol, 3-octanone, and 1-octen-3-one were dominant. All these compounds originate from the enzymatic oxidation of linoleic acid via the lipoxygenase (LOX) pathway [15,18] and are widely recognized as key odor-active contributors to the characteristic fresh mushroom aroma [18,27]. C8 volatiles such as 1-octen-3-ol and 1-octen-3-one are known as major contributors to mushroom-like odor, with 1-octen-3-one showing strong sensory relevance despite its lower abundance [27]. Therefore, the behavior of C8 volatiles provides information not only on biochemical degradation, but also on the preservation of freshness-related aroma potential. The progressive decrease in these compounds over storage reflects enzyme inactivation, lipid substrate depletion, and enhanced volatilization under thermal stress [18]. The declines were more pronounced in STD samples, while SB packaging maintained moderately higher levels of these metabolites up to Days 5 and 8, indicating that its controlled internal atmosphere delayed oxidative degradation.

Secondary metabolites such as 2-ethyl-1-hexanol, butanoic acid, hexanoic acid, and 2-methyl-propanoic acid showed sharp increases over time, particularly in the later stages of storage, and more markedly in STD samples. These compounds are indicators of tissue degradation, microbial activity, and fermentative stress, commonly associated with lipid peroxidation and amino acid catabolism. The aldehydes such as hexanal, heptanal, and benzaldehyde changed during the storage. These changes further reflect the dynamic interplay between oxidative deterioration, enzymatic activity, and volatilization losses during storage [14,18].

At mid-storage (Days 5 and 8), fermentation markers such as acetone, acetoin, and 2,3-butanedione showed mild but consistent changes, indicative of a temporary metabolic shift toward anaerobic respiration under oxygen limitation and RTS conditions [11]. Aromatic and ester compounds including benzyl alcohol, benzeneacetaldehyde, and ethyl butyrate varied irregularly or declined over time, likely due to volatilization losses and disrupted biosynthetic activities [16]. Statistically significant differences were mainly observed between packaging types and storage time, particularly at later stages and under ambient and RTS conditions. Out of 66 VOCs reported in GC, RTS, Table 1 (Table S1), 44 compounds (≈67%) exhibited significant time-dependent changes (*p* < 0.05) in STD samples, compared to 33 compounds (50%) in SB samples. Over time the VOC profile shifted progressively toward degradation-related alcohols, acids, and aldehydes. SB storage slowed this transition by preserving mushroom aromatic compounds and delaying the accumulation of spoilage-associated volatiles. However, long-term storage still led to marked biochemical and oxidative deterioration. Taken together, these data confirm that SB packaging partially preserved the original volatile fingerprint, reducing the breadth of VOC changes and slowing the biochemical transition toward a spoilage-associated profile. Accumulation of compounds such as ethanol, acetaldehyde, methanethiol, organic acids and alcohols related to degradation suggests a transition to fermentative and spoilage-related metabolism during storage, especially in STD samples and under ambient or RTS conditions. However, this study did not carry out microbiological analyses and therefore the possible microbial contribution to these VOC changes cannot be distinguished from the endogenous mushroom metabolism. Thus, microbial involvement is presented as a possible contributor to spoilage-associated VOC formation, but not as a directly quantified mechanism.

**Figure 6:**
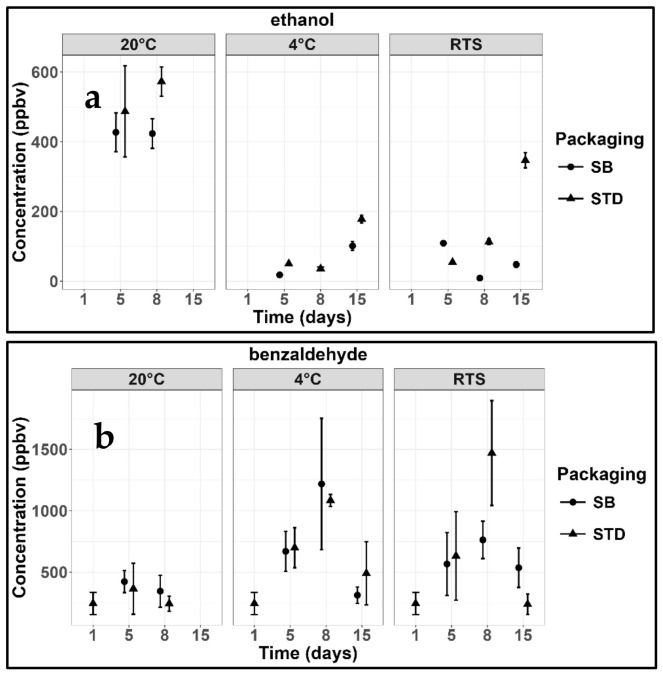
GC–MS boxplots of selected VOCs in *Agaricus bisporus* during storage under different packaging and temperature regimes: (**a**) ethanol and (**b**) benzaldehyde.

To confirm the trends observed in the non-destructive PTR–ToF–MS analysis, Figure 6 reports concentrations of (a) ethanol and (b) benzaldehyde, chosen as representative markers of spoilage and mushroom aroma, respectively. GC–MS showed a sharp increase in ethanol under ambient and RTS conditions, particularly in STD packaging. Statistical analysis confirmed that ethanol concentrations were significantly higher (*p* < 0.05) under ambient and RTS conditions compared to refrigerated storage (see Table 1), with STD samples consistently showing higher values than SB at corresponding time points, confirming its role as a robust spoilage biomarker. Benzaldehyde showed an early increase followed by a decline under RTS and ambient conditions, whereas refrigerated SB samples retained more stable concentrations throughout the storage period. Overall, the GC–MS data were fully consistent with the PTR-ToF-MS fingerprints. Spoilage-related compounds accumulated progressively under suboptimal storage conditions. In contrast, freshness-associated volatiles were better preserved under refrigeration and SB packaging.

According to Supplementary Table S5, storage under refrigerated conditions resulted in relative stability for the majority of mass peaks throughout the 15-day period, reflecting the capability of refrigeration to suppress enzymatic activity, respiration and microbial growth [7]. However, a selected group of 15 key metabolic indicators in Table 2 exhibited significant temporal and treatment-dependent variations. Low-molecular-weight alcohol and carbonyl fragments such as *m*/*z* 31.018, 33.034, 39.023, 41.039, 43.018, and 43.055 were present at low intensities during early storage but increased markedly in later stages, indicating progressive metabolic activation and the onset of oxidative and fermentative reactions as tissues aged. Concurrently, freshness-related aldehydes derived from lipid oxidation—such as *m*/*z* 101.095 (t.i. hexanal or 2-methylpentanal) and *m*/*z* 113.095 (t.i. 2-heptenal)—decreased from Day 1 to Day 15, reflecting a shift in dominant metabolic pathways toward fermentative processes and altered lipid metabolism, in agreement with VOC formation pathways and flavor-evolution mechanisms reported for *A. bisporus* [15,18]. In contrast, butanal (*m*/*z* 73.067) remained relatively stable throughout storage, with no statistically significant differences between packaging systems.

Compared to STD, these changes were attenuated under SB conditions at 4 °C. Compounds such as acetone (*m*/*z* 59.049), 2-propanol (*m*/*z* 61.066), and acetic acid (*m*/*z* 61.029) demonstrated clear late-stage accumulation, revealing localized oxygen limitations and partial anaerobic metabolism [12,13]. Acetic acid in particular, indicative of mild anaerobic metabolism and associated with sour off notes, remained significantly lower and stable up to Day 8 in SB samples, suggesting that smart packaging restrained the accumulation of this fermentative marker. Overall, refrigeration limited volatilome changes over time by preserving low emission rates and delaying both fermentative and oxidative reactions [7], while SB packaging enhanced this stability by moderating gas exchange and humidity, further delaying the accumulation of off-flavor volatiles.

The combined use of PTR-ToF-MS and GC–MS provided a comprehensive picture of VOC evolution during storage. GC–MS identified a total of 67 compounds, of which 23 increased, 29 decreased, and 15 remained approximately stable during shelf life. In the static headspace PTR-ToF-MS dataset, 283 mass peaks were detected, of which 48 were retained after statistical filtering, with 10 increasing, 8 decreasing, and 30 remaining approximately stable. When focusing on key freshness and spoilage markers, both techniques showed consistent trends: shared compounds included ethanol, acetone, acetic acid, hexanal, and 2-heptenal, which in PTR-ToF-MS corresponded to *m*/*z* 47.049, 59.049, 61.029, 101.095, and 113.095. GC–MS additionally resolved the classic C_8_ mushroom aroma compounds 1-octen-3-ol, 3-octanone, and 1-octen-3-one, which cannot be uniquely assigned in PTR-ToF-MS due to overlapping signals. Conversely, PTR-ToF-MS detected low-molecular-weight oxygenated species such as *m*/*z* 31.018 and 33.034 (tentatively associated with methanol) that were not reported in the GC–MS dataset, highlighting the complementary nature of the two analytical approaches.

## 4. Conclusions

This study demonstrates that postharvest quality of *Agaricus bisporus* is governed by tightly coupled physiological and biochemical processes that respond differently to temperature regimes and packaging conditions. Refrigeration remained the primary tool for extending shelf life, suppressing respiration and preserving freshness-associated volatiles such as 1-octen-3-one and benzaldehyde. However, SB packaging provided a crucial and additive protective effect: by establishing a passive modified atmosphere and acting as a physical barrier against desiccation, it stabilized respiratory activity, reduced weight loss, and significantly delayed the accumulation of key spoilage markers (including ethanol, acetaldehyde, and methanethiol) across both dynamic and static headspace measurements. This metabolic buffering effect was most pronounced under repeated temperature stress conditions, where SB-packaged mushrooms exhibited superior homeostasis compared to standard containers, highlighting the practical relevance of smart packaging for real-world cold chain scenarios where thermal fluctuations are unavoidable.

The integrated volatilomics framework employed here, which combines real-time non-destructive PTR-ToF-MS monitoring of intact mushrooms with destructive static headspace analysis by PTR-ToF-MS and GC–MS, enabled the simultaneous tracking of physiological signals and internal biochemical transformations. The consistency between PTR-ToF-MS fingerprints and GC–MS compound-specific data supports VOC-based monitoring as a practical tool for postharvest quality assessment in highly perishable products. Future studies should integrate enzyme activity assays, oxidative damage markers, microbiological analyses, sensory evaluation and consumer-relevant quality metrics to link the observed VOC dynamics with perceived freshness and explore sensor-based decision thresholds for smart logistics applications.

In addition, future work could explore the integration of SB packaging with edible polysaccharide- or protein-based coatings. Such coatings may provide an additional surface-level barrier against moisture loss, gas exchange, enzymatic browning, and surface deterioration, while SB packaging stabilizes the surrounding microenvironment. The combination of coating-based surface protection and smart microenvironmental buffering may further improve mushroom shelf stability under realistic cold-chain conditions.

## Figures and Tables

**Table 1:** Selected VOCs identified by GC–MS analysis under RTS conditions. For each mass peak, the mean ± SD values for each sample are accompanied by Tukey’s homogeneous group letters, indicating significant differences (*p* < 0.05) across the samples.

	Day 1	Day 5		Day 8		Day 15	
Compound	STD	STD	SB	STD	SB	STD	SB
1-octen-3-ol	2251.63 ± 123.67 e	274.15 ± 41.91 abc	326.50 ± 55.64 a	140.18 ± 21.63 bcd	301.18 ± 95.65 ab	98.01 ± 7.63 cd	80.94 ± 6.04 d
1-octen-3-one	276.84 ± 76.87 b	117.78 ± 4.11 ab	86.91 ± 20.87 a	179.16 ± 130.14 ab	149.58 ± 55.57 ab	43.07 ± 13.27 a	52.37 ± 17.10 a
2,3-butanedione		3.86 ± 0.50 bc	3.67 ± 0.62 abc	6.07 ± 1.48 b	0.50 ± 0.19 a	15.06 ± 2.27 d	2.46 ± 0.52 ac
2-ethyl-1-hexanol	4.25 ± 0.46 a	13.67 ± 0.68 a	58.68 ± 4.99 b	142.74 ± 18.27 c	24.65 ± 4.34 a	388.04 ± 19.26 d	226.89 ± 13.05 e
2-methyl propanoic acid	0.20 ± 0.13 d	7.68 ± 0.23 ab	17.44 ± 1.99 c	16.00 ± 3.07 c	1.85 ± 1.11 ad	78.18 ± 5.27 e	13.90 ± 1.34 bc
3-octanone	875.45 ± 26.99 d	126.72 ± 3.46 a	85.08 ± 7.21 ab	93.25 ± 24.30 ab	82.29 ± 12.58 b	24.96 ± 8.82 c	21.99 ± 3.04 c
acetoin		1.92 ± 0.04 d	4.41 ± 0.49 a	6.30 ± 0.81 b		5.89 ± 0.12 b	0.70 ± 0.09 c
acetone	1.40 ± 0.23 b	3.74 ± 0.50 ab	5.19 ± 0.95 abc	7.01 ± 0.70 ac	8.92 ± 2.32 c	6.77 ± 0.80 ac	25.37 ± 2.99 d
benzaldehyde	244.43 ± 90.50 a	631.92 ± 360.56 a	566.46 ± 256.20 a	1470.58 ± 426.92 b	762.55 ± 151.94 a	238.28 ± 83.16 a	536.01 ± 160.60 a
benzeneacetaldehyde	4.98 ± 0.37 a	8.73 ± 2.02 ab	5.65 ± 0.95 a	27.64 ± 11.46 c	13.85 ± 8.62 abc	17.76 ± 3.09 abc	22.57 ± 5.92 bc
benzyl alcohol	177.10 ± 65.07 abc	139.37 ± 29.64 ab	429.60 ± 151.00 ac	198.16 ± 131.16 abc	522.13 ± 265.36 c	62.42 ± 25.98 b	283.69 ± 27.29 abc
ethyl butyrate		0.29 ± 0.08 a	0.86 ± 0.28 a	0.52 ± 0.12 a		8.02 ± 0.82 b	0.37 ± 0.10 a
heptanal	7.77 ± 0.93 bc	5.70 ± 0.38 ab	8.26 ± 0.17 c	4.60 ± 0.65 a	3.81 ± 1.79 a	4.01 ± 0.23 a	4.91 ± 0.38 a
hexanal	293.35 ± 45.63 ab	275.98 ± 17.93 ab	373.38 ± 17.74 a	182.32 ± 61.40 b	180.31 ± 95.89 b	172.22 ± 4.52 b	200.06 ± 31.80 b
hexanoic acid	11.61 ± 2.29 c	5.68 ± 0.90 ab	8.82 ± 0.56 ac	5.44 ± 1.10 ab	4.42 ± 2.43 b	5.17 ± 0.32 ab	5.76 ± 1.25 ab
butanoic acid	0.34 ± 0.17 b	2.54 ± 0.24 ab	11.02 ± 3.55 c	7.43 ± 2.00 ac	2.18 ± 1.30 ab	54.14 ± 4.09 d	5.69 ± 0.32 abc

**Table 2:** Selected key mass peaks tentatively identified by destructive PTR–ToF–MS in *Agaricus bisporus* during refrigerated storage (4 °C). For each mass peak, the mean ± SD values for each sample are accompanied by Tukey’s homogeneous group letters, indicating significant differences (*p* < 0.05) across the samples. Asterisks (*) indicates the tentatively identified compounds employing PTR-ToF-MS that were validated by GC–MS analysis.

*m*/*z*	Formula	Tentative Identification	Day-1	Day-5		Day-8		Day-15	
			STD	STD	SB	STD	SB	STD	SB
31.018	CH3O^+^	Common fragment	2.41 ± 0.04 c	6.79 ± 0.06 a	3.99 ± 0.49 bc	6.07 ± 0.31 ab	1.54 ± 0.01 c	26.07 ± 1.50 d	16.74 ± 1.77 e
33.034	CH5O^+^	methanol	3.65 ± 0.03 bc	5.59 ± 0.04 ab	5.78 ± 0.99 ab	6.71 ± 0.30 a	3.24 ± 0.31 c	15.64 ± 0.87 d	12.43 ± 1.10 e
39.023	C3H3^+^	aromatic/diene/Alkyne fragment	103.86 ± 1.68 d	30.72 ± 3.16 ab	30.92 ± 5.49 ab	34.60 ± 5.37 ab	23.56 ± 0.97 a	50.49 ± 1.80 c	38.61 ± 4.44 b
41.039	C3H5^+^	alkyl fragment	26.55 ± 0.49 c	8.55 ± 1.04 a	8.46 ± 1.46 a	9.86 ± 1.53 a	6.39 ± 0.15 a	17.83 ± 0.93 b	14.37 ± 1.65 b
43.018	C2H3O^+^	acetic acid fragment	5.79 ± 0.69 ac	12.60 ± 1.01 ab	7.91 ± 1.88 ac	15.54 ± 3.63 b	3.62 ± 0.48 c	32.60 ± 3.67 d	19.29 ± 2.91 b
43.055	C3H7^+^	Common fragment	3.49 ± 0.05 ab	2.35 ± 0.13 ab	2.54 ± 0.39 ab	3.65 ± 0.29 a	1.33 ± 0.03 b	10.90 ± 0.78 c	11.36 ± 1.28 c
47.049	C2H7O^+^	ethanol *	0.69 ± 0.09 c	140.68 ± 1.52 a	56.81 ± 8.83 bc	112.12 ± 9.10 ab	0.48 ± 0.22 c	626.21 ± 45.88 d	358.08 ± 46.31 e
49.012	CH5S^+^	methanethiol	1.00 ± 0.06 b	0.41 ± 0.13 a	0.83 ± 0.11 bc	1.00 ± 0.07 b	0.55 ± 0.21 ac	0.63 ± 0.06 ac	0.50 ± 0.02 a
59.049	C3H7O^+^	acetone *	17.55 ± 0.88 a	15.40 ± 0.35 a	27.51 ± 5.03 bc	35.22 ± 2.47 bd	19.66 ± 1.39 ac	37.06 ± 2.44 d	65.81 ± 2.93 e
61.029	C2H5O2^+^	acetic acid *	2.47 ± 0.83 b	6.92 ± 0.27 ab	6.32 ± 2.16 ab	14.33 ± 5.29 ac	2.81 ± 0.91 ab	22.03 ± 4.76 c	13.90 ± 4.82 abc
73.067	C4H9O+	butanal *	1.46 ± 0.22 a	2.40 ± 1.27 a	1.39 ± 0.24 a	2.16 ± 0.33 a	2.19 ± 1.53 a	3.03 ± 0.52 a	2.19 ± 0.68 a
101.095	C6H13O^+^	hexanal/2-methyl pentanal *	28.03 ± 1.66 d	9.39 ± 1.03 ab	8.90 ± 2.16 ab	11.10 ± 2.41 a	5.12 ± 2.84 bc	8.16 ± 0.79 ab	2.93 ± 0.28 c
107.049	C7H7O^+^	benzaldehyde *	3.46 ± 0.24 a	10.38 ± 6.95 a	2.81 ± 0.51 a	4.62 ± 0.45 a	7.40 ± 6.86 a	2.44 ± 1.95 a	3.15 ± 1.54 a
111.113	C8H15+	1-octen-3-ol fragment	16.64 ± 1.32 c	1.29 ± 0.19 ab	1.87 ± 0.31 a	1.26 ± 0.24 ab	1.38 ± 0.21 ab	0.62 ± 0.09 b	0.45 ± 0.04 b
113.095	C7H13O^+^	2-heptenal	2.62 ± 0.38 c	0.84 ± 0.15 a	0.55 ± 0.08 ab	0.60 ± 0.09 ab	0.57 ± 0.21 ab	0.70 ± 0.05 ab	0.37 ± 0.01 b

## Data Availability

Data is available from the corresponding author upon reasonable request.

## Supplementary Materials

The following supporting information can be downloaded at: https://www.mdpi.com/article/10.3390/jof12070477/s1, Figure S1. Representative photographs of *Agaricus bisporus* stored in SB packaging. Figure S2. Representative photographs of *Agaricus bisporus* stored in STD packaging. Table S1. VOCs identified by GC-MS analysis under RTS conditions. Table S2. VOCs identified by GC-MS analysis under refrigerated storage (4 °C). Table S3. VOCs identified by GC-MS analysis under Ambient storage (20 °C). Table S4. Tentatively identified mass peaks from the destructive PTR–ToF–MS analysis in *Agaricus bisporus* RTS conditions. Table S5. Tentatively identified mass peaks from the destructive PTR–ToF–MS analysis in *Agaricus bisporus* during refrigerated storage (4 °C). Table S6. Tentatively identified mass peaks from the destructive PTR–ToF–MS analysis in *Agaricus bisporus* during Ambient temperature (20 °C) storage. Table S7. Tentatively identified mass peaks from the nondestructive PTR–ToF–MS analysis in *Agaricus bisporus* RTS conditions. Table S8. Tentatively identified mass peaks from the nondestructive PTR–ToF–MS analysis in *Agaricus bisporus* during refrigerated storage (4 °C). Table S9. Tentatively identified mass peaks from the nondestructive PTR–ToF–MS analysis in *Agaricus bisporus* during Ambient temperature (20 °C) storage.
