# Supplementary material for: Impact of Temperature Regimes and Smart Packaging on Volatilome Evolution During the Shelf Life of Agaricus bisporus
Source: J Fungi (Basel). 2026 Jun 30;12(7):477. doi: 10.3390/jof12070477 (PMC13413179; doi:10.3390/jof12070477)
Supplement: Supplementary file 1 [file jof-12-00477-s001.zip › jof-4327628-supplementary.pdf]

Table S1. VOCs identified by GC-MS analysis under RTS conditions. For each mass peak, the mean ± SD values for each sample are accompanied by Tukey's homogeneous group letters, indicating significant differences ($p < 0.05$) across the samples.

	day-1	day-5		day-8		day-15	
compounds	STD-1	STD-5	SB-5	STD-8	SB-8	STD-15	SB-15
(E)-2-octen-1-ol	53.39 ± 4.61 c	9.39 ± 1.15 ab	11.87 ± 2.86 a	7.68 ± 2.39 ab	12.05 ± 3.31 a	2.94 ± 0.24 b	3.02 ± 0.19 b
1-dodecanol	1.13 ± 0.28 a	2.80 ± 1.95 a	3.11 ± 1.79 a	1.60 ± 1.07 a	0.75 ± 0.06 a	0.76 ± 0.02 a	1.16 ± 0.18 a
1-hepten-3-ol	7.66 ± 0.56 b	0.69 ± 0.11 a	0.87 ± 0.15 a	0.55 ± 0.17 a	0.96 ± 0.46 a	0.26 ± 0.01 a	
1-nonen-3-ol	1.57 ± 0.00 d	0.20 ± 0.04 a	0.39 ± 0.06 b		0.33 ± 0.05 bc		0.27 ± 0.05 ac
1-octanol	3.29 ± 0.20 b	0.76 ± 0.12 a	0.93 ± 0.05 a	1.08 ± 0.13 a	1.05 ± 0.52 a		0.58 ± 0.07 a
1-octen-3-ol	2251.63 ± 123.67 e	274.15 ± 41.91 abc	326.50 ± 55.64 a	140.18 ± 21.63 bcd	301.18 ± 95.65 ab	98.01 ± 7.63 cd	80.94 ± 6.04 d
1-octen-3-one	276.84 ± 76.87 b	117.78 ± 4.11 ab	86.91 ± 20.87 a	179.16 ± 130.14 ab	149.58 ± 55.57 ab	43.07 ± 13.27 a	52.37 ± 17.10 a
2,3-butanediol				1.09 ± 0.29 a		3.60 ± 2.03 a	1.30 ± 0.57 a
2,3-butanedione		3.86 ± 0.50 bc	3.67 ± 0.62 abc	6.07 ± 1.48 b	0.50 ± 0.19 a	15.06 ± 2.27 d	2.46 ± 0.52 ac
2,4-decadienal	4.21 ± 0.92 b	2.20 ± 0.19 a	2.10 ± 0.94 a	2.56 ± 0.75 ab	1.54 ± 0.12 a	1.63 ± 0.22 a	1.61 ± 0.32 a
2,4-nonadienal	17.23 ± 1.53 b	8.87 ± 0.64 a	9.98 ± 3.59 a	9.60 ± 2.30 a	9.12 ± 1.12 a	7.02 ± 0.58 a	7.43 ± 1.51 a
2,7-octanedione?	15.03 ± 2.51 d	8.62 ± 0.55 a	10.09 ± 1.53 a	4.96 ± 0.75 bc	2.18 ± 1.12 b	6.59 ± 0.38 ac	4.40 ± 0.49 bc
2-butyl furan	6.14 ± 2.14 b	2.43 ± 0.31 a	4.50 ± 0.81 ab	2.39 ± 0.87 a	1.60 ± 1.17 a	1.62 ± 0.37 a	2.43 ± 0.68 a
2-ethyl-1-hexanol	4.25 ± 0.46 a	13.67 ± 0.68 a	58.68 ± 4.99 b	142.74 ± 18.27 c	24.65 ± 4.34 a	388.04 ± 19.26 d	226.89 ± 13.05 e
2-ethyl-hexanoic acid	1.26 ± 0.08 b	4.12 ± 1.76 ab	4.70 ± 0.91 ab	80.54 ± 18.55 c	32.32 ± 20.62 a	24.41 ± 2.04 ab	124.04 ± 7.20 d

2-heptenal	34.11 ± 17.59 a	14.51 ± 2.09 a	17.97 ± 7.24 a	21.55 ± 14.43 a	30.59 ± 15.50 a	13.25 ± 3.66 a	15.47 ± 3.93 a
2-hexenal	1.34 ± 0.26 a	0.83 ± 0.18 a	1.20 ± 0.20 a	1.12 ± 0.45 a	1.33 ± 0.37 a	0.83 ± 0.07 a	0.96 ± 0.14 a
2-methyl butanal	0.65 ± 0.20 a	2.64 ± 1.84 a	1.85 ± 0.98 a	10.24 ± 4.10 b	4.84 ± 1.87 a	1.95 ± 0.62 a	4.74 ± 0.68 a
2-methyl butanoic acid		3.69 ± 0.25 b	7.07 ± 1.00 a	6.10 ± 1.41 ab		27.91 ± 1.81 c	3.84 ± 0.33 b
2-methyl pentanal?	42.65 ± 8.59 abc	47.20 ± 3.56 ab	55.80 ± 6.56 a	35.19 ± 2.71 bc	29.66 ± 10.97 c	25.58 ± 1.96 c	32.77 ± 3.30 bc
2-methyl propanoic acid	0.20 ± 0.13 d	7.68 ± 0.23 ab	17.44 ± 1.99 c	16.00 ± 3.07 c	1.85 ± 1.11 ad	78.18 ± 5.27 e	13.90 ± 1.34 bc
2-methyl-1-butanol		1.48 ± 0.11 b	6.99 ± 0.21 a	6.24 ± 0.50 a	0.69 ± 0.23 b	42.44 ± 2.78 c	5.49 ± 0.25 a
2-methyl-3-octanone	4.14 ± 0.39 ab	3.76 ± 0.44 a	5.17 ± 0.07 b	4.03 ± 0.78 ab	4.31 ± 0.41 ab	2.22 ± 0.19 c	3.40 ± 0.28 a
2-nonenal	3.36 ± 1.62 a	1.87 ± 0.06 a	2.14 ± 0.62 a	2.06 ± 0.48 a	2.24 ± 0.67 a	2.10 ± 0.48 a	2.14 ± 0.49 a
2-octanone	1.77 ± 0.36 b	2.81 ± 1.27 ab	4.63 ± 1.33 ab	9.92 ± 5.45 a	8.71 ± 4.19 ab	2.67 ± 0.27 ab	3.77 ± 0.41 ab
2-pentyl furan	54.53 ± 17.91 bc	33.84 ± 2.98 abc	57.05 ± 11.64 b	23.57 ± 8.35 a	16.61 ± 13.64 a	24.03 ± 4.81 a	26.66 ± 4.39 ac
2-propanol		1.37 ± 0.08 c		3.68 ± 0.40 a		8.79 ± 0.38 b	10.53 ± 1.34 b
2-undecanone	17.36 ± 1.04 c	10.46 ± 0.91 a	22.94 ± 4.46 b	7.94 ± 1.72 a	10.08 ± 0.82 a	8.33 ± 0.88 a	10.33 ± 0.86 a
3,3-dimethyl-butanamide?	3.51 ± 0.14 b	1.35 ± 0.08 ab	21.97 ± 0.34 c	1.00 ± 0.07 a	6.73 ± 1.16 d	1.31 ± 0.05 ab	21.49 ± 1.91 c
3,4-dihydro-6-methyl-2H-pyran?	34.62 ± 5.19 a	26.12 ± 1.99 abc	31.94 ± 2.64 ab	23.19 ± 2.30 bc	18.61 ± 4.84 c	19.24 ± 1.63 c	20.80 ± 2.08 c
3-(methylthio)-1-propanol	0.27 ± 0.06 a	0.21 ± 0.07 a	0.36 ± 0.06 ab	0.79 ± 0.20 b	0.47 ± 0.26 ab	0.61 ± 0.25 ab	0.44 ± 0.24 ab
3-ethyl-2-methyl-1,3-hexadiene	7.41 ± 1.57 c	4.41 ± 0.59 ab	5.98 ± 0.61 ac	4.60 ± 0.49 ab	4.18 ± 1.25 ab	2.81 ± 0.43 b	4.16 ± 0.32 ab
3-heptanone	2.18 ± 0.13 d	0.33 ± 0.07 a	0.47 ± 0.05 ab	0.71 ± 0.18 bc	0.57 ± 0.11 ab	0.57 ± 0.05 ab	0.98 ± 0.21 c

3-methyl butanal	0.80 ± 0.22 a	2.09 ± 1.17 a	1.56 ± 0.85 a	8.19 ± 3.81 b	3.72 ± 1.62 ab	1.55 ± 0.42 a	3.87 ± 0.99 ab
3-methyl hexanal	10.41 ± 1.73 c	7.12 ± 0.35 ab	9.02 ± 1.56 ac	4.72 ± 0.37 bd	2.69 ± 1.12 d	4.58 ± 0.38 bd	4.55 ± 0.38 bd
3-methyl-2-butanone	15.57 ± 1.20 ab	14.24 ± 0.41 ab	16.77 ± 2.17 a	11.62 ± 1.38 b	11.70 ± 2.91 b	6.99 ± 0.73 c	13.44 ± 1.17 ab
3-octanol	103.14 ± 2.78 c	6.71 ± 0.84 a	6.81 ± 2.01 a	3.27 ± 0.66 ab	4.83 ± 1.68 ab	1.06 ± 0.23 b	0.82 ± 0.16 b
3-octanone	875.45 ± 26.99 d	126.72 ± 3.46 a	85.08 ± 7.21 ab	93.25 ± 24.30 ab	82.29 ± 12.58 b	24.96 ± 8.82 c	21.99 ± 3.04 c
3-phenylpropanol		1.15 ± 0.03 a	2.36 ± 0.12 ab	2.79 ± 0.20 ab	0.23 ± 0.05 a	28.34 ± 2.42 c	3.93 ± 0.48 b
6-methyl-5-hepten-2-one	1.31 ± 0.25 a	1.21 ± 0.13 a	1.22 ± 0.05 a	1.92 ± 0.19 b	0.93 ± 0.16 a	4.71 ± 0.27 c	0.98 ± 0.06 a
acetic acid	1.26 ± 0.35 b	8.15 ± 0.94 ab	12.59 ± 1.83 a	8.79 ± 1.57 ab	2.25 ± 0.82 b	31.11 ± 3.40 c	51.69 ± 7.65 d
acetoin		1.92 ± 0.04 d	4.41 ± 0.49 a	6.30 ± 0.81 b		5.89 ± 0.12 b	0.70 ± 0.09 c
acetone	1.40 ± 0.23 b	3.74 ± 0.50 ab	5.19 ± 0.95 abc	7.01 ± 0.70 ac	8.92 ± 2.32 c	6.77 ± 0.80 ac	25.37 ± 2.99 d
benzaldehyde	244.43 ± 90.50 a	631.92 ± 360.56 a	566.46 ± 256.20 a	1470.58 ± 426.92 b	762.55 ± 151.94 a	238.28 ± 83.16 a	536.01 ± 160.60 a
benzeneacetaldehyde	4.98 ± 0.37 a	8.73 ± 2.02 ab	5.65 ± 0.95 a	27.64 ± 11.46 c	13.85 ± 8.62 abc	17.76 ± 3.09 abc	22.57 ± 5.92 bc
benzyl alcohol	177.10 ± 65.07 abc	139.37 ± 29.64 ab	429.60 ± 151.00 ac	198.16 ± 131.16 abc	522.13 ± 265.36 c	62.42 ± 25.98 b	283.69 ± 27.29 abc
butanal	0.20 ± 0.01 a	0.45 ± 0.13 ab	0.67 ± 0.25 ab	0.82 ± 0.32 ab	0.87 ± 0.43 ab	0.26 ± 0.00 a	1.21 ± 0.39 b
butanoic acid	0.34 ± 0.17 b	2.54 ± 0.24 ab	11.02 ± 3.55 c	7.43 ± 2.00 ac	2.18 ± 1.30 ab	54.14 ± 4.09 d	5.69 ± 0.32 abc
decanoic acid	0.64 ± 0.10 a	0.59 ± 0.08 a	0.68 ± 0.21 a	0.36 ± 0.16 a	1.57 ± 1.65 a	0.61 ± 0.13 a	0.55 ± 0.23 a
diethyl phthalate	2.27 ± 0.25 a	208.43 ± 178.75 a	168.27 ± 179.63 a	3.41 ± 0.93 a	0.64 ± 0.83 a	2.05 ± 0.60 a	1.92 ± 1.14 a

ethanol		54.46 ± 1.25 d	109.12 ± 3.82 a	113.44 ± 8.51 a	9.07 ± 2.00 b	346.96 ± 21.86 c	47.87 ± 7.46 d
ethyl butyrate		0.29 ± 0.08 a	0.86 ± 0.28 a	0.52 ± 0.12 a		8.02 ± 0.82 b	0.37 ± 0.10 a
ethyl isobutyrate			0.58 ± 0.01 a	0.31 ± 0.02 a		2.45 ± 0.25 b	
heptanal	7.77 ± 0.93 bc	5.70 ± 0.38 ab	8.26 ± 0.17 c	4.60 ± 0.65 a	3.81 ± 1.79 a	4.01 ± 0.23 a	4.91 ± 0.38 a
hexanal	293.35 ± 45.63 ab	275.98 ± 17.93 ab	373.38 ± 17.74 a	182.32 ± 61.40 b	180.31 ± 95.89 b	172.22 ± 4.52 b	200.06 ± 31.80 b
hexanoic acid	11.61 ± 2.29 c	5.68 ± 0.90 ab	8.82 ± 0.56 ac	5.44 ± 1.10 ab	4.42 ± 2.43 b	5.17 ± 0.32 ab	5.76 ± 1.25 ab
hexanol	129.04 ± 5.18 ac	63.83 ± 9.09 a	138.99 ± 13.50 abc	172.79 ± 45.18 bc	210.01 ± 52.47 b	80.90 ± 4.56 a	121.95 ± 3.97 ac
linalool		0.34 ± 0.04 c		0.75 ± 0.11 a		1.72 ± 0.16 b	
methional	0.48 ± 0.18 a	1.13 ± 0.42 a	1.22 ± 0.49 a	1.44 ± 0.69 a	0.76 ± 0.78 a	0.67 ± 0.24 a	1.30 ± 0.39 a
methyl-2-ethylhexanoate?		0.16 ± 0.00 b	0.41 ± 0.03 ab	0.76 ± 0.19 c	0.30 ± 0.09 ab	0.51 ± 0.02 ac	0.78 ± 0.13 c
nonanal	3.95 ± 0.30 a	2.70 ± 0.18 a	4.10 ± 0.47 a	2.04 ± 0.18 a	4.83 ± 5.47 a	2.28 ± 0.14 a	1.93 ± 0.30 a
nonanoic acid	2.59 ± 0.36 a	4.80 ± 2.52 a	4.87 ± 3.38 a	1.37 ± 0.64 a	4.43 ± 3.42 a	3.41 ± 1.52 a	2.95 ± 1.44 a
octanal	2.60 ± 0.23 a	1.49 ± 0.09 a	2.72 ± 0.10 a	1.07 ± 0.05 a	2.12 ± 2.09 a	1.24 ± 0.19 a	1.14 ± 0.18 a
octanoic acid	0.61 ± 0.37 a	0.55 ± 0.27 a	0.55 ± 0.49 a	0.36 ± 0.03 a	0.76 ± 0.71 a	0.65 ± 0.40 a	0.63 ± 0.40 a
pentanal	19.52 ± 1.75 bc	15.98 ± 1.22 ab	23.01 ± 0.56 c	11.21 ± 1.55 ad	9.99 ± 4.64 d	8.48 ± 0.68 d	13.63 ± 0.93 ad
pentyl oxirane	2.96 ± 1.59 a	2.78 ± 0.64 a	3.07 ± 1.02 a	2.47 ± 0.34 a	2.99 ± 0.56 a	1.49 ± 0.67 a	2.94 ± 0.49 a

Table S2. VOCs identified by GC-MS analysis under refrigerated storage (4 °C). For each mass peak, the mean ± SD values for each sample are accompanied by Tukey's homogeneous group letters, indicating significant differences ($p < 0.05$) across the samples.

compounds	day1	day-5		day-8		day-15	
	STD	STD	SB	STD	SB	STD	SB
(E)-2-octen-1-ol	53.39 ± 4.61 c	8.66 ± 1.25 a	7.09 ± 0.99 ab	10.18 ± 3.08 a	6.02 ± 1.20 ab	3.98 ± 0.48 ab	2.37 ± 0.39 b
1-dodecanol	1.13 ± 0.28 a	0.76 ± 0.32 a	3.32 ± 1.34 b	0.35 ± 0.04 a	0.91 ± 0.24 a	1.07 ± 0.61 a	1.08 ± 0.24 a
1-hepten-3-ol	7.66 ± 0.56 c	0.73 ± 0.16 ab	1.25 ± 0.17 a	0.87 ± 0.30 ab	0.89 ± 0.05 ab	0.32 ± 0.04 b	
1-nonen-3-ol	1.57 ± 0.00 e	0.23 ± 0.03 ab	0.37 ± 0.06 c	0.18 ± 0.03 ad	0.33 ± 0.01 c	0.10 ± 0.03 d	0.29 ± 0.02 bc
1-octanol	3.29 ± 0.20 b	0.62 ± 0.07 a	0.49 ± 0.10 a	0.74 ± 0.14 a	0.44 ± 0.05 a	0.50 ± 0.01 a	
1-octen-3-ol	2251.63 ± 123.67 d	288.10 ± 53.79 abc	452.47 ± 63.92 a	310.75 ± 81.28 ab	241.42 ± 54.33 bc	136.45 ± 1.85 bc	103.12 ± 14.11 c
1-octen-3-one	276.84 ± 76.87 c	111.00 ± 58.58 ab	175.23 ± 62.79 ac	130.77 ± 34.61 ab	157.94 ± 27.95 abc	48.46 ± 17.86 ab	38.12 ± 12.18 b
2,3-butanediol						0.98 ± 0.12 a	1.00 ± 0.11 a
2,3-butanedione		3.64 ± 1.07 b	1.21 ± 0.26 a	3.76 ± 0.92 b		7.65 ± 1.10 c	5.11 ± 0.47 b
2,4-decadienal	4.21 ± 0.92 b	1.66 ± 1.01 a	1.06 ± 0.40 a	1.04 ± 0.22 a	1.11 ± 0.34 a	1.37 ± 0.83 a	0.97 ± 0.22 a
2,4-nonadienal	17.23 ± 1.53 b	6.49 ± 3.53 a	3.66 ± 1.16 a	5.77 ± 0.84 a	4.39 ± 0.79 a	6.08 ± 2.74 a	4.30 ± 0.46 a
2,7-octanedione?	15.03 ± 2.51 e	8.97 ± 1.45 a	4.02 ± 0.93 bc	5.38 ± 0.83 bd	1.54 ± 0.06 c	7.76 ± 0.78 ad	2.29 ± 0.12 bc
2-butyl furan	6.14 ± 2.14 b	2.83 ± 0.59 a	1.75 ± 0.62 a	2.18 ± 0.66 a	1.21 ± 0.32 a	2.62 ± 0.49 a	1.53 ± 0.32 a
2-ethyl-1-hexanol	4.25 ± 0.46 b	62.27 ± 9.46 ab	45.74 ± 3.88 ab	74.51 ± 8.17 ab	57.63 ± 3.63 ab	116.85 ± 4.96 a	597.86 ± 75.23 c
2-ethyl-hexanoic acid	1.26 ± 0.08 a	5.33 ± 0.78 a	7.04 ± 0.48 a	58.16 ± 12.56 b	142.23 ± 16.65 c	13.54 ± 1.83 a	224.65 ± 24.05 d
2-heptenal	34.11 ± 17.59 b	12.07 ± 8.63 ab	8.83 ± 4.39 a	12.07 ± 4.22 ab	9.67 ± 2.57 a	9.58 ± 3.87 a	10.07 ± 2.72 a
2-hexenal	1.34 ± 0.26 b	0.65 ± 0.17 a	0.47 ± 0.11 a	0.69 ± 0.08 a	0.58 ± 0.11 a	0.70 ± 0.23 a	0.55 ± 0.10 a

2-methyl butanal	0.65 ± 0.20 a	2.86 ± 0.88 a	3.65 ± 1.74 ab	5.25 ± 1.00 ab	9.09 ± 4.95 b	2.70 ± 1.29 a	2.50 ± 0.48 a
2-methyl butanoic acid		3.12 ± 0.38 a	4.23 ± 0.72 a	3.43 ± 0.71 a		13.21 ± 1.48 b	7.16 ± 0.53 c
2-methyl pentanal?	42.65 ± 8.59 a	45.59 ± 5.61 a	21.34 ± 2.35 b	36.15 ± 5.39 a	15.27 ± 0.34 b	36.86 ± 3.47 a	15.15 ± 1.11 b
2-methyl propanoic acid	0.20 ± 0.13 c	6.15 ± 1.39 ab	6.73 ± 1.77 a	9.00 ± 2.53 a	0.89 ± 0.64 bc	45.61 ± 2.89 d	25.28 ± 2.88 e
2-methyl-1-butanol		2.54 ± 0.07 a	1.65 ± 0.10 a	1.91 ± 0.14 a		18.80 ± 0.98 b	10.71 ± 1.05 c
2-methyl-3-octanone	4.14 ± 0.39 b	3.35 ± 0.55 ab	1.97 ± 0.15 cd	3.11 ± 0.41 a	2.19 ± 0.06 cd	2.81 ± 0.04 ac	1.77 ± 0.15 d
2-nonenal	3.36 ± 1.62 b	1.60 ± 0.62 ab	1.38 ± 0.33 a	1.39 ± 0.21 a	1.31 ± 0.12 a	1.75 ± 0.25 ab	1.59 ± 0.13 ab
2-octanone	1.77 ± 0.36 a	2.59 ± 0.69 a	3.76 ± 0.84 a	4.29 ± 0.88 a	8.64 ± 2.90 b	3.20 ± 0.57 a	2.75 ± 0.40 a
2-pentyl furan	54.53 ± 17.91 b	39.44 ± 5.62 ab	24.98 ± 7.75 ac	23.41 ± 6.97 ac	13.19 ± 3.05 c	34.26 ± 8.01 abc	17.20 ± 4.18 ac
2-propanol		1.08 ± 0.04 a		2.59 ± 0.13 a		6.27 ± 1.17 b	5.12 ± 0.78 b
2-undecanone	17.36 ± 1.04 c	12.31 ± 0.20 a	7.54 ± 0.94 b	7.70 ± 1.08 b	4.58 ± 0.53 b	11.65 ± 2.37 a	6.56 ± 1.09 b
3,3-dimethyl-butanamide?	3.51 ± 0.14 a	3.57 ± 0.51 a	17.33 ± 1.76 b	1.95 ± 0.25 a	9.54 ± 0.62 c	1.74 ± 0.11 a	22.68 ± 2.74 d
3,4-dihydro-6-methyl-2H-pyran?	34.62 ± 5.19 b	26.77 ± 4.78 ab	14.27 ± 1.17 cd	21.82 ± 1.34 ac	10.22 ± 0.16 d	24.75 ± 2.51 a	10.44 ± 0.24 d
3-(methylthio)-1-propanol	0.27 ± 0.06 a	0.31 ± 0.13 a	0.23 ± 0.08 a	0.57 ± 0.20 a	0.24 ± 0.15 a		
3-ethyl-2-methyl-1,3-hexadiene	7.41 ± 1.57 b	3.73 ± 0.88 a	2.57 ± 0.13 a	3.86 ± 0.47 a	2.36 ± 0.35 a	3.30 ± 0.52 a	2.18 ± 0.04 a
3-heptanone	2.18 ± 0.13 c	0.46 ± 0.04 a	1.09 ± 0.10 b	0.52 ± 0.09 a	1.52 ± 0.40 b	0.36 ± 0.04 a	1.59 ± 0.28 b
3-methyl butanal	0.80 ± 0.22 a	2.25 ± 0.46 a	2.90 ± 1.24 ab	4.07 ± 0.81 ab	7.10 ± 3.59 b	2.03 ± 1.02 a	2.20 ± 0.49 a
3-methyl hexanal	10.41 ± 1.73 e	7.13 ± 1.38 a	3.74 ± 0.62 bcd	4.78 ± 0.57 abc	1.74 ± 0.14 d	5.65 ± 0.51 ac	2.52 ± 0.31 bd

3-methyl-2-butanone	15.57 ± 1.20 b	13.68 ± 2.07 ab	7.41 ± 0.81 c	12.19 ± 0.60 a	5.65 ± 0.27 c	11.86 ± 1.44 a	6.53 ± 0.91 c
3-octanol	103.14 ± 2.78 d	5.46 ± 0.77 a	48.21 ± 5.19 b	3.62 ± 1.08 a	17.97 ± 3.05 c	2.12 ± 0.14 a	7.75 ± 1.16 a
3-octanone	875.45 ± 26.99 c	98.13 ± 12.63 a	381.62 ± 41.33 b	81.63 ± 13.39 a	303.09 ± 57.19 b	28.33 ± 3.85 a	75.30 ± 8.63 a
3-phenylpropanol		1.21 ± 0.06 a	1.67 ± 0.24 a	1.24 ± 0.17 a		14.92 ± 0.56 b	9.44 ± 1.28 c
6-methyl-5-hepten-2-one	1.31 ± 0.25 a	1.44 ± 0.22 a	0.64 ± 0.11 bc	1.07 ± 0.12 ab	0.64 ± 0.05 bc	2.45 ± 0.16 d	0.63 ± 0.10 c
acetic acid	1.26 ± 0.35 b	11.53 ± 1.35 a	4.99 ± 1.51 ab	6.82 ± 1.84 ab	2.37 ± 0.50 b	61.92 ± 6.77 c	36.24 ± 3.72 d
acetoin		1.83 ± 0.17 b	0.26 ± 0.04 a	1.59 ± 0.22 b		3.09 ± 0.30 c	0.97 ± 0.10 d
acetone	1.40 ± 0.23 b	3.60 ± 0.72 ab	5.23 ± 0.69 a	5.52 ± 0.93 a	4.57 ± 1.83 ab	6.07 ± 1.60 a	10.52 ± 1.38 c
benzaldehyde	244.43 ± 90.50 c	699.15 ± 162.95 abc	669.59 ± 162.68 abc	1084.27 ± 48.62 ab	1218.54 ± 534.96 b	490.48 ± 257.56 ac	312.20 ± 66.66 c
benzeneacetaldehyde	4.98 ± 0.37 a	13.49 ± 4.04 a	14.88 ± 14.71 a	12.85 ± 8.01 a	17.38 ± 3.66 a	5.67 ± 1.02 a	10.11 ± 3.63 a
benzyl alcohol	177.10 ± 65.07 a	167.45 ± 77.52 a	169.15 ± 107.07 a	288.47 ± 117.36 a	179.30 ± 48.32 a	147.33 ± 21.07 a	132.15 ± 58.84 a
butanal	0.20 ± 0.01 b	0.49 ± 0.19 ab	0.50 ± 0.13 ab	0.84 ± 0.24 ab	1.06 ± 0.47 a	0.60 ± 0.21 ab	0.78 ± 0.19 ab
butanoic acid	0.34 ± 0.17 a	4.21 ± 1.39 a	2.86 ± 1.38 a	4.07 ± 2.17 a	1.31 ± 1.17 a	26.09 ± 2.42 b	14.22 ± 2.23 c
decanoic acid	0.64 ± 0.10 a	0.76 ± 0.32 a	0.83 ± 0.14 a	0.58 ± 0.20 a	0.55 ± 0.12 a	0.66 ± 0.33 a	0.69 ± 0.15 a
diethyl phthalate	2.27 ± 0.25 a	2.18 ± 1.06 a	243.61 ± 156.69 b		6.07 ± 3.80 a	2.89 ± 0.70 a	2.34 ± 0.46 a
ethanol		50.11 ± 3.45 b	18.23 ± 2.44 a	35.85 ± 5.06 ab		178.15 ± 10.82 c	101.34 ± 12.88 d
ethyl butyrate		0.36 ± 0.13 a		0.28 ± 0.05 a		2.63 ± 0.37 b	0.91 ± 0.01 c
ethyl isobutyrate						1.39 ± 0.24 b	0.46 ± 0.14 a

heptanal	7.77 ± 0.93 b	6.24 ± 0.82 ab	3.45 ± 0.26 cd	4.63 ± 1.00 ac	2.72 ± 0.14 d	5.32 ± 0.28 a	3.02 ± 0.12 cd
hexanal	293.35 ± 45.63 a	287.74 ± 29.66 a	126.04 ± 25.04 b	219.78 ± 39.91 ac	106.09 ± 25.29 b	235.51 ± 14.14 ac	156.50 ± 9.77 bc
hexanoic acid	11.61 ± 2.29 b	5.40 ± 0.63 a	4.19 ± 0.29 a	4.91 ± 0.76 a	2.81 ± 0.70 a	5.48 ± 0.64 a	3.19 ± 0.32 a
hexanol	129.04 ± 5.18 b	60.64 ± 11.31 a	48.71 ± 11.95 a	125.16 ± 16.91 b	58.72 ± 6.98 a	61.27 ± 12.91 a	47.27 ± 6.32 a
linalool		0.54 ± 0.15 a		0.88 ± 0.13 a		1.69 ± 0.14 b	
methional	0.48 ± 0.18 b	1.60 ± 0.54 ab	1.40 ± 0.21 ab	1.30 ± 0.48 ab	2.56 ± 1.42 a	0.86 ± 0.32 ab	0.82 ± 0.17 ab
methyl-2-ethylhexanoate		0.32 ± 0.04 ac	0.16 ± 0.04 a	0.92 ± 0.21 b	0.52 ± 0.11 c	0.55 ± 0.08 c	0.92 ± 0.13 b
nonanal	3.95 ± 0.30 d	2.73 ± 0.81 a	1.66 ± 0.06 bc	1.57 ± 0.12 bc	1.05 ± 0.18 b	2.31 ± 0.12 ac	1.27 ± 0.15 b
nonanoic acid	2.59 ± 0.36 a	3.48 ± 2.41 a	7.90 ± 2.16 b	3.50 ± 0.75 ab	2.51 ± 0.81 a	3.14 ± 2.15 a	2.89 ± 1.02 a
octanal	2.60 ± 0.23 d	1.52 ± 0.33 a	1.23 ± 0.06 ab	0.95 ± 0.04 bc	0.70 ± 0.07 c	1.23 ± 0.13 ab	0.64 ± 0.06 c
octanoic acid	0.61 ± 0.37 a	0.41 ± 0.27 a	0.94 ± 0.45 a	0.51 ± 0.07 a	0.39 ± 0.11 a	0.38 ± 0.11 a	0.38 ± 0.10 a
pentanal	19.52 ± 1.75 b	17.30 ± 2.16 ab	9.22 ± 1.29 cd	12.14 ± 2.33 ce	7.05 ± 0.35 d	14.24 ± 0.56 ae	8.70 ± 0.55 cd
pentyl oxirane	2.96 ± 1.59 a	3.16 ± 1.90 a	1.79 ± 0.63 a	2.76 ± 0.39 a	1.53 ± 0.41 a	1.85 ± 0.40 a	1.56 ± 0.48 a

Table S3. VOCs identified by GC-MS analysis under Ambient storage (20 °C). For each mass peak, the mean ± SD values for each sample are accompanied by Tukey's homogeneous group letters, indicating significant differences ($p < 0.05$) across the samples.

COMPOUND	day-1	day-5		day-8	
	STD	STD	SB	STD	SB
(E)-2-octen-1-ol	53.39 ± 4.61 b	7.02 ± 2.94 a	3.47 ± 0.91 a	1.91 ± 0.23 a	2.10 ± 0.34 a

1-dodecanol	1.13 ± 0.28 a	1.16 ± 0.69 a	3.34 ± 0.82 b	0.52 ± 0.19 a	0.85 ± 0.07 a
1-hepten-3-ol	7.66 ± 0.56 b	0.65 ± 0.22 a		0.26 ± 0.03 a	
1-nonen-3-ol	1.57 ± 0.00 c		0.25 ± 0.03 a		0.31 ± 0.01 b
1-octanol	3.29 ± 0.20				
1-octen-3-ol	2251.63 ± 123.67 b	182.72 ± 68.02 a	90.48 ± 14.60 a	58.47 ± 12.81 a	63.12 ± 7.30 a
1-octen-3-one	276.84 ± 76.87 b	86.33 ± 57.09 a	59.10 ± 32.16 a	28.76 ± 13.53 a	32.80 ± 17.50 a
2,3-butanediol		4.52 ± 3.34 a	3.88 ± 2.61 a	3.64 ± 0.37 a	2.92 ± 0.36 a
2,3-butanedione		15.84 ± 3.31 a	12.78 ± 2.67 a	14.11 ± 3.43 a	11.02 ± 3.43 a
2,4-decadienal	4.21 ± 0.92 b	1.87 ± 1.32 a	2.22 ± 0.10 ab	0.75 ± 0.38 a	0.92 ± 0.26 a
2,4-nonadienal	17.23 ± 1.53 b	8.14 ± 4.71 a	9.28 ± 0.88 a	4.47 ± 1.52 a	5.24 ± 0.94 a
2,7-octanedione	15.03 ± 2.51 b	6.07 ± 1.76 a	6.68 ± 0.24 a	3.19 ± 0.73 a	3.62 ± 0.41 a
2-butyl furan	6.14 ± 2.14 b	1.72 ± 0.31 a	2.18 ± 0.85 a	1.40 ± 0.42 a	1.63 ± 0.47 a
2-ethyl-1-hexanol	4.25 ± 0.46 e	77.08 ± 19.05 a	241.88 ± 27.77 b	346.17 ± 37.88 c	633.50 ± 29.91 d
2-ethyl-hexanoic acid	1.26 ± 0.08 e	10.78 ± 1.37 a	17.63 ± 3.20 b	39.01 ± 1.89 c	54.55 ± 3.72 d
2-heptenal	34.11 ± 17.59 a	22.38 ± 18.70 a	24.89 ± 14.04 a	10.55 ± 4.22 a	12.28 ± 4.51 a
2-hexenal	1.34 ± 0.26 a	0.92 ± 0.56 a	1.16 ± 0.28 a	0.89 ± 0.20 a	0.74 ± 0.18 a
2-methyl butanal	0.65 ± 0.20 a	1.96 ± 0.89 a	2.64 ± 1.03 a	1.23 ± 0.26 a	2.01 ± 0.92 a
2-methyl butanoic acid		40.79 ± 9.25 ac	31.15 ± 0.61 a	63.41 ± 3.87 b	51.42 ± 1.65 bc
2-methyl pentanal?	42.65 ± 8.59 b	31.53 ± 10.23 ab	34.24 ± 1.94 ab	14.30 ± 2.79 c	17.84 ± 2.46 ac

2-methyl propanoic acid	0.20 ± 0.13 c	166.72 ± 41.02 ab	117.91 ± 2.16 a	206.80 ± 16.37 b	164.57 ± 15.10 ab
2-methyl-1-butanol		27.46 ± 7.34 a	23.85 ± 3.43 a	49.87 ± 4.90 b	33.28 ± 3.06 a
2-methyl-3-octanone	4.14 ± 0.39 b	3.33 ± 1.21 ab	3.56 ± 0.17 ab	2.26 ± 0.45 a	2.44 ± 0.33 a
2-nonenal	3.36 ± 1.62 a	3.04 ± 1.95 a	3.03 ± 1.22 a	2.03 ± 0.48 a	1.82 ± 0.43 a
2-octanone	1.77 ± 0.36 a	3.50 ± 1.59 a	8.28 ± 1.25 b	3.12 ± 0.71 a	4.56 ± 0.94 a
2-pentyl furan	54.53 ± 17.91 b	25.23 ± 3.50 a	29.38 ± 10.90 ab	15.03 ± 4.38 a	18.66 ± 5.26 a
2-propanol		24.58 ± 7.77 b	31.74 ± 5.95 ab	47.56 ± 3.83 ac	56.95 ± 6.47 c
2-undecanone	17.36 ± 1.04 c	10.92 ± 3.66 ab	15.80 ± 1.10 ac	7.52 ± 0.94 b	11.93 ± 0.94 ab
3,3-dimethyl-butanamide?	3.51 ± 0.14 a	0.72 ± 0.22 a	21.48 ± 2.71 b	0.30 ± 0.09 a	24.98 ± 1.95 b
3,4-dihydro-6-methyl-2H-pyran?	34.62 ± 5.19 a	24.25 ± 7.71 abc	27.78 ± 2.29 ab	14.52 ± 2.18 c	17.52 ± 1.39 bc
3-(methylthio)-1-propanol	0.27 ± 0.06 a	0.27 ± 0.16 a	0.58 ± 0.08 ab	0.38 ± 0.17 ab	0.88 ± 0.43 b
3-ethyl-2-methyl-1,3-hexadiene	7.41 ± 1.57 c	3.23 ± 0.98 ab	4.52 ± 0.37 a	1.90 ± 0.51 b	2.37 ± 0.10 ab
3-heptanone	2.18 ± 0.13 c	0.32 ± 0.01 a	1.05 ± 0.07 b	0.62 ± 0.16 ab	2.06 ± 0.43 c
3-methyl butanal	0.80 ± 0.22 a	1.67 ± 0.89 a	2.05 ± 0.80 a	0.86 ± 0.23 a	1.37 ± 0.57 a
3-methyl hexanal	10.41 ± 1.73 b	4.48 ± 1.01 a	5.10 ± 0.21 a	2.63 ± 0.47 a	2.88 ± 0.71 a
3-methyl-2-butanone	15.57 ± 1.20 b	10.24 ± 3.73 ab	11.80 ± 2.28 ab	8.67 ± 1.22 ac	4.54 ± 0.18 c
3-octanol	103.14 ± 2.78 c	3.08 ± 1.07 ab	2.25 ± 0.43 a	6.06 ± 0.62 b	3.84 ± 0.19 ab
3-octanone	875.45 ± 26.99 b	65.48 ± 16.74 a	37.73 ± 7.33 a	57.56 ± 6.10 a	51.88 ± 2.13 a
3-phenylpropanol		5.56 ± 1.51 a	8.58 ± 1.44 a	14.11 ± 1.28 b	20.62 ± 1.08 c

6-methyl-5-hepten-2-one	1.31 ± 0.25 a	1.62 ± 0.31 ab	1.85 ± 0.28 ab	1.44 ± 0.16 a	2.19 ± 0.22 b
acetic acid	1.26 ± 0.35 d	77.64 ± 9.00 a	41.57 ± 8.26 b	186.85 ± 20.44 c	95.51 ± 12.08 a
acetoin		19.14 ± 5.14 bc	12.39 ± 1.58 ab	21.25 ± 1.65 c	11.12 ± 0.85 a
acetone	1.40 ± 0.23 b	21.37 ± 5.37 a	21.50 ± 1.33 a	27.52 ± 2.98 a	26.34 ± 2.65 a
benzaldehyde	244.43 ± 90.50 a	364.79 ± 207.62 a	423.15 ± 89.70 a	243.34 ± 61.27 a	344.63 ± 130.16 a
benzeneacetaldehyde	4.98 ± 0.37 a	11.53 ± 7.32 a	14.45 ± 5.03 a	5.14 ± 1.52 a	7.55 ± 3.01 a
benzyl alcohol	177.10 ± 65.07 a	225.47 ± 142.08 a	352.06 ± 200.48 a	162.10 ± 27.94 a	232.29 ± 28.55 a
butanal	0.20 ± 0.01 b	0.41 ± 0.03 ab	0.48 ± 0.07 ab	0.65 ± 0.15 a	0.68 ± 0.20 a
butanoic acid	0.34 ± 0.17 e	49.61 ± 10.19 a	90.69 ± 5.18 b	133.89 ± 14.88 c	219.98 ± 23.54 d
decanoic acid	0.64 ± 0.10 a	1.91 ± 1.75 a	0.60 ± 0.08 a	2.25 ± 1.01 a	0.53 ± 0.19 a
diethyl phthalate	2.27 ± 0.25 a	2.17 ± 1.23 a	151.50 ± 55.60 b		1.65 ± 0.79 a
ethanol		487.20 ± 130.66 a	427.20 ± 55.71 a	572.71 ± 41.91 a	423.62 ± 42.47 a
ethyl butyrate		9.34 ± 2.59 c	26.47 ± 1.38 a	23.90 ± 3.14 a	53.08 ± 6.76 b
ethyl isobutyrate		7.94 ± 2.05 a	7.87 ± 0.53 a	7.07 ± 0.99 a	8.73 ± 1.28 a
heptanal	7.77 ± 0.93 b	4.69 ± 1.38 a	5.23 ± 0.40 a	3.23 ± 0.88 a	3.31 ± 0.51 a
hexanal	293.35 ± 45.63 b	162.87 ± 70.20 a	177.51 ± 23.63 a	106.79 ± 26.57 a	124.93 ± 27.66 a
hexanoic acid	11.61 ± 2.29 a	8.61 ± 1.45 ab	9.02 ± 1.09 a	4.99 ± 0.93 b	4.74 ± 1.10 b
hexanol	129.04 ± 5.18 a	138.40 ± 36.24 a	209.66 ± 32.01 b	76.94 ± 16.68 a	110.23 ± 26.99 a
methional	0.48 ± 0.18 a	0.19 ± 0.09 a	0.35 ± 0.11 a	0.25 ± 0.08 a	0.45 ± 0.22 a

methyl-2-ethylhexanoate?		0.52 ± 0.05 c	1.10 ± 0.06 a	1.18 ± 0.16 a	3.21 ± 0.10 b
nonanal	3.95 ± 0.30 a	2.83 ± 1.35 a	3.32 ± 0.17 a	2.30 ± 0.71 a	2.01 ± 0.45 a
nonanoic acid	2.59 ± 0.36 a	8.21 ± 9.00 a	5.25 ± 0.12 a	8.07 ± 3.90 a	1.67 ± 0.65 a
octanal	2.60 ± 0.23 c	1.43 ± 0.44 ab	1.78 ± 0.08 a	0.95 ± 0.27 b	1.02 ± 0.18 b
octanoic acid	0.61 ± 0.37 a	0.87 ± 0.66 a	0.83 ± 0.22 a	0.70 ± 0.19 a	0.38 ± 0.01 a
pentanal	19.52 ± 1.75 b	9.84 ± 4.80 a	10.77 ± 1.00 a	6.22 ± 1.88 a	7.38 ± 1.67 a
pentyl oxirane	2.96 ± 1.59 a	1.77 ± 1.48 a	2.57 ± 0.98 a	1.32 ± 0.52 a	1.55 ± 0.43 a

Table S4. Tentatively identified mass peaks from the destructive PTR–ToF–MS analysis in *Agaricus bisporus* RTS conditions. For each mass peak, the mean ± SD values for each sample are accompanied by Tukey's homogeneous group letters, indicating significant differences ($p < 0.05$) across the samples.

m/z	Formula	tentative identification	day-1	day-5		day-8		day-15	
			STD-1	STD-5	SB-5	STD-8	SB-8	STD-15	SB-15
31.018	CH3O+	Unspecific fragment	2.41 ± 0.04 d	9.34 ± 0.33 a	15.26 ± 0.60 b	18.76 ± 0.88 c	4.12 ± 0.08 d	62.14 ± 0.68 e	12.34 ± 0.62 f
33.034	CH5O+	methanol	3.65 ± 0.03 c	7.88 ± 0.29 a	8.92 ± 0.38 ab	9.56 ± 0.51 b	4.01 ± 0.10 c	17.25 ± 0.20 d	11.37 ± 0.64 e
39.023	C3H3+	aromatic / diene / Alkyne fragment	103.86 ± 1.68 c	38.17 ± 7.40 ab	54.96 ± 19.11 a	37.01 ± 1.68 ab	26.47 ± 3.56 b	92.81 ± 5.94 c	45.44 ± 0.68 ab
41.039	C3H5+	alkyl fragment	26.55 ± 0.49 e	10.75 ± 2.04 ab	16.13 ± 4.48 ac	12.74 ± 0.54 abc	7.87 ± 1.38 b	34.86 ± 1.26 d	17.44 ± 0.59 c
43.018	C2H3O+	acetic acid fragment	5.79 ± 0.69 c	16.51 ± 0.22 a	26.90 ± 1.50 b	19.66 ± 0.60 a	6.53 ± 0.19 c	45.47 ± 3.08 d	20.46 ± 0.83 a
43.055	C3H7+	Common fragment	3.49 ± 0.05 a	3.34 ± 0.38 a	5.95 ± 0.60 b	7.60 ± 0.47 c	3.70 ± 0.28 a	20.26 ± 0.54 d	17.22 ± 0.85 e
47.049	C2H7O+	ethanol*	0.69 ± 0.09 d	203.65 ± 12.91 a	355.27 ± 22.29 b	447.68 ± 25.75 c	30.68 ± 1.61 d	1550.45 ± 21.29 e	154.09 ± 8.90 f
49.012	CH5S+	methanethiol	1.00 ± 0.06 b	0.58 ± 0.11 ab	0.80 ± 0.38 ab	0.35 ± 0.06 a	0.50 ± 0.08 ab	0.42 ± 0.04 a	0.44 ± 0.09 a

49.03	CH5O2+	methanediol	0.00 ± 0.00 d	0.24 ± 0.02 a	0.39 ± 0.08 b	0.55 ± 0.04 c	0.07 ± 0.00 d	1.84 ± 0.03 e	0.28 ± 0.02 a
53.039	C4H5+	Common fragment (propargyl /allyl-type)	1.97 ± 0.04 ab	2.36 ± 0.55 ab	3.16 ± 1.77 a	1.31 ± 0.15 ab	1.37 ± 0.13 ab	1.21 ± 0.17 ab	1.10 ± 0.09 b
55.052	C4H7+	alkene / cycloalkene / terpene / dehydrated alcohol fragment	36.94 ± 1.72 c	13.31 ± 4.11 ab	20.27 ± 15.78 ac	1.73 ± 1.36 b	4.90 ± 0.98 ab	3.55 ± 1.39 ab	1.97 ± 1.59 ab
57.035	C3H5O+	acrolein fragment/ acrolein (2-propenal).	7.71 ± 0.54 d	3.21 ± 0.56 ab	4.42 ± 1.12 ac	2.67 ± 0.14 b	2.27 ± 0.24 b	4.76 ± 0.21 c	2.58 ± 0.03 b
57.07	C4H9+	butanol	3.03 ± 0.06 ac	5.27 ± 0.22 a	14.94 ± 0.32 b	15.30 ± 0.52 b	2.42 ± 0.17 c	95.06 ± 2.06 d	8.85 ± 0.32 e
59.049	C3H7O+	acetone*	17.55 ± 0.88 b	26.70 ± 0.70 ab	31.60 ± 2.75 a	48.45 ± 1.74 c	67.28 ± 2.11 d	47.06 ± 2.11 c	182.58 ± 10.00 e
61.029	C2H5O2+	acetic acid*	2.47 ± 0.83 a	6.76 ± 1.19 ab	9.35 ± 5.35 abc	4.63 ± 0.32 a	3.67 ± 0.72 a	12.61 ± 3.31 bc	15.38 ± 1.27 c
61.066	C3H9O+	2-propanol*	0.03 ± 0.01 a	0.03 ± 0.01 a	0.08 ± 0.06 a	0.24 ± 0.02 b	0.12 ± 0.01 a	0.68 ± 0.04 c	0.65 ± 0.06 c
63.044	C2H7O2+	Cluster of acetaldehyde	1.07 ± 0.01 d	8.99 ± 0.43 ab	15.27 ± 3.35 c	12.55 ± 0.39 ac	1.71 ± 0.11 d	26.88 ± 0.75 e	5.19 ± 0.74 bd
69.071	C5H9+	Isoprene/ 3-hexen-2-ol	22.02 ± 0.92 b	8.36 ± 2.72 a	10.20 ± 5.71 a	5.33 ± 0.63 a	5.51 ± 0.45 a	3.66 ± 0.52 a	4.58 ± 0.26 a
71.014	C3H3O2+	fragment (common from acids/esters)	0.07 ± 0.01 d	0.04 ± 0.00 abc	0.05 ± 0.01 ad	0.04 ± 0.00 ab	0.02 ± 0.00 c	0.13 ± 0.00 e	0.03 ± 0.00 bc
71.05	C4H7O+	2-butenal / methyl vinyl ketone (MVK)	1.87 ± 0.13 a	1.16 ± 0.27 abc	1.48 ± 0.74 ab	0.64 ± 0.10 bc	0.58 ± 0.15 c	1.24 ± 0.18 abc	0.55 ± 0.08 c
71.086	C5H11+	methyl butanol/ pentanol	3.06 ± 0.09 cd	1.67 ± 0.29 ab	2.40 ± 0.57 ac	1.71 ± 0.14 ab	1.43 ± 0.26 b	3.32 ± 0.16 d	1.60 ± 0.10 b
73.067	C4H9O+	butanal*	1.46 ± 0.22 b	2.37 ± 0.72 ab	3.11 ± 1.09 abc	4.19 ± 0.51 ac	2.69 ± 0.48 abc	4.29 ± 0.64 c	3.32 ± 0.74 abc
75.048	C3H7O2+	propanoic acid, hydroxyacetone (acetol),	0.22 ± 0.01 ab	0.20 ± 0.04 ab	0.19 ± 0.03 ab	0.17 ± 0.04 ab	0.13 ± 0.02 a	0.36 ± 0.04 c	0.22 ± 0.02 b

		and methyl acetate							
77.023	C2H5O3+	2-Hydroxyacetic acid (glycolic acid)	0.89 ± 0.15 a	0.95 ± 0.20 a	0.80 ± 0.09 a	1.03 ± 0.15 a	0.97 ± 0.24 a	1.02 ± 0.30 a	0.93 ± 0.07 a
77.06	C3H9O2+	Propylene glycol	0.14 ± 0.01 b	0.20 ± 0.02 ab	0.22 ± 0.01 abc	0.31 ± 0.01 cd	0.40 ± 0.02 d	0.29 ± 0.00 ac	1.05 ± 0.08 e
79.053	C6H7+	benzene	2.47 ± 0.25 a	4.25 ± 2.11 a	4.02 ± 1.96 a	6.78 ± 1.67 a	3.91 ± 0.26 a	3.29 ± 0.61 a	4.11 ± 1.35 a
81.067	C6H9+	Monoterpene fragment	0.94 ± 0.05 c	0.43 ± 0.09 ab	0.57 ± 0.25 a	0.28 ± 0.01 ab	0.28 ± 0.00 ab	0.29 ± 0.04 ab	0.23 ± 0.02 b
85.068	C5H9O+	2-pentenal / 1-penten-3-one	0.51 ± 0.01 c	0.25 ± 0.06 ab	0.29 ± 0.14 a	0.14 ± 0.01 ab	0.14 ± 0.02 ab	0.17 ± 0.02 ab	0.12 ± 0.01 b
85.101	C6H13+	hexanol	1.47 ± 0.27 cd	0.91 ± 0.28 abc	1.53 ± 0.23 d	0.89 ± 0.12 ab	1.37 ± 0.15 bcd	0.90 ± 0.05 abc	0.79 ± 0.10 a
89.06	C4H9O2+	acetoin /butanoic acid/ isobutyric acid */ ethyl acetate	0.20 ± 0.03 b	0.61 ± 0.04 ab	1.31 ± 0.49 c	0.90 ± 0.12 ac	0.25 ± 0.06 b	3.42 ± 0.29 d	0.90 ± 0.12 ac
91.056	C4H11S+	butanethiol isomers (1-/2-/iso-/tert-)	1.07 ± 0.28 a	1.10 ± 0.30 a	2.09 ± 0.91 a	0.99 ± 0.20 a	1.54 ± 0.40 a	1.11 ± 0.15 a	1.60 ± 0.19 a
91.076	$C4H11O2^+$	2,3–butanediol*	0.00 ± 0.00 a	0.09 ± 0.01 a	0.31 ± 0.06 b	0.36 ± 0.02 b	0.00 ± 0.00 a	3.02 ± 0.10 c	0.02 ± 0.01 a
93.037	C6H5O+	Phenolic / Aromatic fragment	2.19 ± 0.01 ab	2.30 ± 0.06 ab	2.51 ± 0.37 a	1.95 ± 0.09 b	2.50 ± 0.17 a	2.46 ± 0.13 a	2.38 ± 0.14 ab
97.066	C6H9O+	Cyclohexenone / 2,4-Hexadienal	0.11 ± 0.00 c	0.06 ± 0.01 ab	0.08 ± 0.03 ac	0.04 ± 0.01 b	0.04 ± 0.01 ab	0.04 ± 0.00 b	0.04 ± 0.00 b
101.095	C6H13O+	hexanal/ 2-methyl pentanal*	28.03 ± 1.66 b	12.59 ± 3.51 ab	17.85 ± 12.67 ab	4.41 ± 0.97 a	5.58 ± 1.47 a	5.23 ± 1.22 a	4.31 ± 0.97 a
103.08	C5H11O2+	2-methyl butanoic acid*	0.13 ± 0.01 ab	0.09 ± 0.01 ab	0.13 ± 0.07 ab	0.07 ± 0.01 a	0.06 ± 0.01 a	0.17 ± 0.02 b	0.05 ± 0.00 a
105.09	C5H13O2+	1,2-Pentanediol	0.34 ± 0.02 b	0.17 ± 0.04 a	0.22 ± 0.11 ab	0.11 ± 0.02 a	0.11 ± 0.02 a	0.12 ± 0.02 a	0.10 ± 0.01 a
107.049	C7H7O+	benzaldehyde*	3.46 ± 0.24 b	6.71 ± 3.81 ab	5.64 ± 3.49 ab	11.56 ± 3.04 a	5.75 ± 0.36 ab	5.10 ± 1.18 ab	5.70 ± 2.31 ab

111.044	C6H7O2+	2-Furancarboxaldehyde, 5-methyl- (C6H6O2)	0.05 ± 0.01 c	0.11 ± 0.00 ab	0.12 ± 0.01 a	0.10 ± 0.01 b	0.12 ± 0.01 ab	0.11 ± 0.01 ab	0.11 ± 0.00 ab
111.113	C8H15+		16.64 ± 1.32 c	1.43 ± 0.11 a	1.20 ± 0.18 ab	0.90 ± 0.11 ab	1.21 ± 0.23 ab	0.50 ± 0.06 ab	0.32 ± 0.05 b
113.095	C7H13O+	2-heptenal	2.62 ± 0.38 c	1.16 ± 0.25 a	1.84 ± 0.42 b	0.60 ± 0.09 a	0.91 ± 0.03 a	0.69 ± 0.08 a	0.58 ± 0.08 a
117.091	C6H1302+	ethyl isobutyrate or hexanoic acid or ethyl butyrate*	0.05 ± 0.00 ab	0.05 ± 0.00 ab	0.10 ± 0.01 a	0.09 ± 0.01 ab	0.03 ± 0.00 b	0.67 ± 0.06 c	0.05 ± 0.00 ab
121.066	C8H9O+	benzeneacetaldehyde (phenylacetaldehyde)*	0.06 ± 0.01 a	0.15 ± 0.03 abc	0.12 ± 0.04 ab	0.21 ± 0.04 cd	0.14 ± 0.01 abc	0.24 ± 0.03 d	0.18 ± 0.01 bcd
133.057			0.00 ± 0.00 a	0.01 ± 0.00 a	0.01 ± 0.00 a	0.03 ± 0.00 bc	0.03 ± 0.01 b	0.02 ± 0.00 c	0.04 ± 0.00 d
139.044	C7H7O3+	hydroxybenzoic acid isomers: salicylic (o-HBA), m-HBA, p-HBA	0.00 ± 0.00 a	0.00 ± 0.00 a	0.00 ± 0.00 a	0.01 ± 0.00 a	0.00 ± 0.00 a	0.01 ± 0.00 a	0.01 ± 0.00 a
143.11	C8H15O2+	Hexenyl acetate isomers (e.g., 3-hexen-1-yl acetate)	0.08 ± 0.01 a	0.05 ± 0.01 ab	0.08 ± 0.03 a	0.04 ± 0.01 b	0.06 ± 0.00 ab	0.03 ± 0.01 b	0.04 ± 0.00 b

Table S5. Tentatively identified mass peaks from the destructive PTR–ToF–MS analysis in *Agaricus bisporus* during refrigerated storage (4 °C). For each mass peak, the mean ± SD values for each sample are accompanied by Tukey's homogeneous group letters, indicating significant differences ($p < 0.05$) across the samples.

m/z	Formula	tentative identification	day-1	day-5		day-8		day-15	
			STD	STD	SB	STD	SB	STD	SB
31.018	CH3O+	Unspecific fragment	2.41 ± 0.04 c	6.79 ± 0.06 a	3.99 ± 0.49 bc	6.07 ± 0.31 ab	1.54 ± 0.01 c	26.07 ± 1.50 d	16.74 ± 1.77 e

33.034	CH5O+	methanol	3.65 ± 0.03 bc	5.59 ± 0.04 ab	5.78 ± 0.99 ab	6.71 ± 0.30 a	3.24 ± 0.31 c	15.64 ± 0.87 d	12.43 ± 1.10 e
39.023	C3H3+	aromatic / diene / Alkyne fragment	103.86 ± 1.68 d	30.72 ± 3.16 ab	30.92 ± 5.49 ab	34.60 ± 5.37 ab	23.56 ± 0.97 a	50.49 ± 1.80 c	38.61 ± 4.44 b
41.039	C3H5+	alkyl fragment	26.55 ± 0.49 c	8.55 ± 1.04 a	8.46 ± 1.46 a	9.86 ± 1.53 a	6.39 ± 0.15 a	17.83 ± 0.93 b	14.37 ± 1.65 b
43.018	C2H3O +	acetic acid fragment	5.79 ± 0.69 ac	12.60 ± 1.01 ab	7.91 ± 1.88 ac	15.54 ± 3.63 b	3.62 ± 0.48 c	32.60 ± 3.67 d	19.29 ± 2.91 b
43.055	C3H7+	Common fragment	3.49 ± 0.05 ab	2.35 ± 0.13 ab	2.54 ± 0.39 ab	3.65 ± 0.29 a	1.33 ± 0.03 b	10.90 ± 0.78 c	11.36 ± 1.28 c
47.049	C2H7O +	ethanol*	0.69 ± 0.09 c	140.68 ± 1.52 a	56.81 ± 8.83 bc	112.12 ± 9.10 ab	0.48 ± 0.22 c	626.21 ± 45.88 d	358.08 ± 46.31 e
49.012	CH5S+	methanethiol	1.00 ± 0.06 b	0.41 ± 0.13 a	0.83 ± 0.11 bc	1.00 ± 0.07 b	0.55 ± 0.21 ac	0.63 ± 0.06 ac	0.50 ± 0.02 a
49.03	CH5O2 +.	methanediol	0.00 ± 0.00 b	0.17 ± 0.00 a	0.05 ± 0.01 b	0.09 ± 0.01 ab	0.00 ± 0.01 b	0.72 ± 0.06 c	0.45 ± 0.05 d
53.039	C4H5+	Common fragment (propargyl/allyl -type)	1.97 ± 0.04 a	2.04 ± 0.37 a	1.82 ± 0.31 a	2.04 ± 0.34 a	1.39 ± 0.24 ab	1.62 ± 0.04 a	0.86 ± 0.06 b
55.052	C4H7+	alkene / cycloalkene / terpene / dehydrated alcohol fragment	36.94 ± 1.72 d	9.43 ± 1.26 ab	7.85 ± 2.03 ab	9.82 ± 3.19 a	2.78 ± 3.93 bc	7.23 ± 0.83 ab	0.28 ± 0.49 c
57.035	C3H5O +	acrolein fragment/ acrolein (2-propenal).	7.71 ± 0.54 b	2.61 ± 0.36 a	2.29 ± 0.40 a	2.33 ± 0.34 a	2.11 ± 0.14 a	3.01 ± 0.11 a	2.20 ± 0.22 a
57.07	C4H9+	butanol fragment / dehydrated alcohol fragment	3.03 ± 0.06 a	4.77 ± 0.21 a	3.28 ± 0.51 a	4.40 ± 0.41 a	1.04 ± 0.09 a	31.17 ± 2.37 b	20.66 ± 2.82 c
59.049	C3H7O +	acetone*	17.55 ± 0.88 a	15.40 ± 0.35 a	27.51 ± 5.03 bc	35.22 ± 2.47 bd	19.66 ± 1.39 ac	37.06 ± 2.44 d	65.81 ± 2.93 e
61.029	C2H5O 2+	acetic acid*	2.47 ± 0.83 b	6.92 ± 0.27 ab	6.32 ±	14.33 ± 5.29 ac	2.81 ± 0.91 ab	22.03 ± 4.76 c	13.90 ± 4.82 abc

					2.16 ab				
61.066	C3H9O +	2-propanol*	0.03 ± 0.01 a	0.00 ± 0.00 a	0.02 ± 0.01 a	0.00 ± 0.00 a	0.01 ± 0.00 a	0.21 ± 0.01 b	0.32 ± 0.09 b
63.044	C2H7O 2+	Cluster of acetaldehyde	1.07 ± 0.01 d	5.59 ± 0.50 ab	2.15 ± 0.45 cd	4.22 ± 0.43 ac	0.65 ± 0.22 d	13.30 ± 1.36 e	6.96 ± 1.59 b
69.071	C5H9+	Isoprene/ 3-hexen-2-ol	22.02 ± 0.92 c	7.01 ± 1.46 a	6.00 ± 1.46 ab	7.30 ± 1.45 a	5.48 ± 0.93 ab	5.26 ± 0.21 ab	2.80 ± 0.64 b
71.014	C3H3O 2+	fragment (common from acids/esters)	0.07 ± 0.01 d	0.03 ± 0.01 ab	0.03 ± 0.01 ab	0.03 ± 0.01 ab	0.02 ± 0.00 a	0.05 ± 0.00 cd	0.04 ± 0.00 bc
71.05	C4H7O +	2-butenal / methyl vinyl ketone (MVK)	1.87 ± 0.13 d	0.86 ± 0.04 ab	0.75 ± 0.16 ac	1.02 ± 0.17 ab	0.46 ± 0.19 c	1.10 ± 0.07 b	0.50 ± 0.02 c
71.086	C5H11+	methyl butanol/ pentanol	3.06 ± 0.09 d	1.29 ± 0.11 ab	1.07 ± 0.16 a	1.77 ± 0.25 bc	1.21 ± 0.05 a	1.81 ± 0.06 c	1.95 ± 0.24 c
73.067	C4H9O +	butanal*	1.46 ± 0.22 a	2.40 ± 1.27 a	1.39 ± 0.24 a	2.16 ± 0.33 a	2.19 ± 1.53 a	3.03 ± 0.52 a	2.19 ± 0.68 a
75.048	C3H7O 2+	propanoic acid, hydroxyaceton e (acetol), and methyl acetate	0.22 ± 0.01 a	0.17 ± 0.00 a	0.16 ± 0.02 a	0.20 ± 0.04 a	0.12 ± 0.01 a	0.23 ± 0.08 a	0.20 ± 0.01 a
77.023	C2H5O 3+	2-Hydroxyacetic acid (glycolic acid)	0.89 ± 0.15 a	1.12 ± 0.12 a	1.01 ± 0.14 a	0.90 ± 0.17 a	1.02 ± 0.01 a	1.05 ± 0.09 a	0.93 ± 0.24 a
77.06	C3H9O 2+	Propylene glycol	0.14 ± 0.01 ab	0.12 ± 0.02 a	0.18 ± 0.04 ab	0.22 ± 0.01 b	0.15 ± 0.04 ab	0.22 ± 0.02 b	0.38 ± 0.03 c
79.053	C6H7+	benzene	2.47 ± 0.25 a	7.03 ± 5.01 a	2.04 ± 0.28 a	3.18 ± 0.21 a	4.63 ± 3.90 a	2.16 ± 1.10 a	2.44 ± 0.92 a
81.067	C6H9+	Monoterpene fragment	0.94 ± 0.05 c	0.39 ± 0.05 a	0.35 ± 0.05 a	0.40 ± 0.07 a	0.28 ± 0.05 ab	0.33 ± 0.01 a	0.19 ± 0.01 b
85.068	C5H9O +	2-pentenal / 1-penten-3-one	0.51 ± 0.01 d	0.19 ± 0.01 ab	0.19 ± 0.03 ab	0.23 ± 0.03 a	0.15 ± 0.01 bc	0.17 ± 0.01 b	0.09 ± 0.01 c
85.101	C6H13+	hexanol	1.47 ± 0.27 b	0.50 ± 0.07 a	0.38 ± 0.10 a	1.08 ± 0.22 b	0.45 ± 0.05 a	0.45 ± 0.07 a	0.37 ± 0.04 a

89.06	C4H9O 2+	acetoin /butanoic acid/ isobutyric acid */ ethyl acetate	0.20 ± 0.03 a	0.51 ± 0.08 a	0.55 ± 0.23 a	1.28 ± 0.39 b	0.20 ± 0.11 a	2.69 ± 0.26 c	1.46 ± 0.16 b
91.056	C4H11S +	butanethiol isomers (1-/2- /iso-/tert-)	1.07 ± 0.28 a	1.25 ± 0.26 a	0.55 ± 0.15 a	0.72 ± 0.15 a	0.96 ± 0.51 a	0.93 ± 0.30 a	1.06 ± 0.25 a
91.076	C4H11 $O2^{+}$	2,3−butanediol *	0.00 ± 0.00 a	0.00 ± 0.00 a	0.01 ± 0.01 a	0.02 ± 0.02 a	0.00 ± 0.00 a	0.57 ± 0.09 b	0.18 ± 0.06 c
93.037	C6H5O +	Phenolic / Aromatic fragment	2.19 ± 0.01 a	2.41 ± 0.04 a	2.34 ± 0.13 a	2.07 ± 0.21 a	2.16 ± 0.20 a	2.36 ± 0.04 a	2.38 ± 0.10 a
97.066	C6H9O +	Cyclohexenone / 2,4- Hexadienal	0.11 ± 0.00 c	0.06 ± 0.01 a	0.05 ± 0.02 ab	0.06 ± 0.02 a	0.04 ± 0.00 ab	0.05 ± 0.00 ab	0.03 ± 0.00 b
101.095	C6H13 O+	hexanal/ 2- methyl pentanal*	28.03 ± 1.66 d	9.39 ± 1.03 ab	8.90 ± 2.16 ab	11.10 ± 2.41 a	5.12 ± 2.84 bc	8.16 ± 0.79 ab	2.93 ± 0.28 c
103.08	C5H11 O2+	2-methyl butanoic acid*	0.13 ± 0.01 d	0.07 ± 0.01 ab	0.07 ± 0.01 abc	0.11 ± 0.03 cd	0.04 ± 0.02 a	0.11 ± 0.01 bcd	0.05 ± 0.01 a
105.09	C5H13 O2+	1,2-Pentanediol	0.34 ± 0.02 c	0.12 ± 0.01 ab	0.11 ± 0.03 ab	0.15 ± 0.04 a	0.11 ± 0.01 ab	0.12 ± 0.00 ab	0.07 ± 0.01 b
107.049	C7H7O +	benzaldehyde*	3.46 ± 0.24 a	10.38 ± 6.95 a	2.81 ± 0.51 a	4.62 ± 0.45 a	7.40 ± 6.86 a	2.44 ± 1.95 a	3.15 ± 1.54 a
111.044	C6H7O 2+	5- methylfurfural / 5-methyl-2- furancarboxald ehyde	0.05 ± 0.01 b	0.11 ± 0.01 a	0.12 ± 0.00 a	0.10 ± 0.01 a	0.10 ± 0.01 a	0.11 ± 0.00 a	0.11 ± 0.01 a
111.113	C8H15+	1-octen-3-ol	16.64 ± 1.32 c	1.29 ± 0.19 ab	1.87 ± 0.31 a	1.26 ± 0.24 ab	1.38 ± 0.21 ab	0.62 ± 0.09 b	0.45 ± 0.04 b
113.095	C7H13 O+	2-heptenal	2.62 ± 0.38 c	0.84 ± 0.15 a	0.55 ± 0.08 ab	0.60 ± 0.09 ab	0.57 ± 0.21 ab	0.70 ± 0.05 ab	0.37 ± 0.01 b
117.091	C6H13 O2+	ethyl isobutyrate or hexanoic acid or ethyl butyrate*	0.05 ± 0.00 b	0.04 ± 0.00 ab	0.04 ± 0.01 ab	0.05 ± 0.00 ab	0.03 ± 0.01 a	0.19 ± 0.01 c	0.10 ± 0.01 d
121.066	C8H9O +	benzeneacetald ehyde (phenylacetald ehyde)*	0.06 ± 0.01 a	0.13 ± 0.03 a	0.07 ± 0.01 a	0.13 ± 0.01 a	0.17 ± 0.09 a	0.14 ± 0.03 a	0.14 ± 0.04 a

133.057	C5H9O4+	Glutaric acid (pentanedioic acid)	0.00 ± 0.00 a	0.01 ± 0.00 a	0.01 ± 0.00 a	0.02 ± 0.00 b	0.03 ± 0.00 c	0.02 ± 0.00 b	0.03 ± 0.00 c
139.044	C7H7O3+	hydroxybenzoic acid isomers: salicylic (o-HBA), m-HBA, p-HBA	0.00 ± 0.00 a	0.00 ± 0.00 a	0.00 ± 0.00 a	0.00 ± 0.00 a	0.00 ± 0.00 a	0.00 ± 0.00 a	0.00 ± 0.00 a
143.11	C8H15O2+	Hexenyl acetate isomers (e.g., 3-hexen-1-yl acetate)	0.08 ± 0.01 c	0.05 ± 0.01 ab	0.03 ± 0.01 ab	0.05 ± 0.01 a	0.05 ± 0.00 ab	0.04 ± 0.01 ab	0.03 ± 0.01 b

Table S6. Tentatively identified mass peaks from the destructive PTR–ToF–MS analysis in *Agaricus bisporus* during Ambient temperature (20 °C) storage. For each mass peak, the mean ± SD values for each sample are accompanied by Tukey's homogeneous group letters, indicating significant differences ($p < 0.05$) across the samples.

m/z	Formula	tentative identification	Day 1	day-5		day-8	
			STD	STD	SB	STD	SB
31.018	CH3O+	Unspecific fragment	2.35 ± 0.11 d	92.38 ± 5.14 a	72.64 ± 1.21 b	115.47 ± 10.07 c	99.23 ± 5.18 a
33.034	CH5O+	methanol	3.61 ± 0.07 c	21.99 ± 1.20 a	19.93 ± 0.77 a	27.53 ± 3.05 b	30.83 ± 1.52 b
39.023	C3H3+	aromatic / diene / Alkyne fragment	101.36 ± 4.49 a	139.25 ± 7.02 a	142.39 ± 3.73 a	209.83 ± 31.82 b	245.79 ± 19.63 b
41.039	C3H5+	alkyl fragment	25.83 ± 1.31 d	55.20 ± 3.41 a	57.31 ± 1.50 a	85.04 ± 11.35 b	101.31 ± 7.38 c
43.018	C2H3O+	acetic acid fragment	5.80 ± 0.49 c	92.53 ± 5.16 a	58.66 ± 20.16 b	35.06 ± 0.39 b	85.02 ± 3.05 a
43.055	C3H7+	Common fragment	3.48 ± 0.04 b	50.55 ± 3.35 a	46.11 ± 15.48 a	26.73 ± 0.25 ab	131.20 ± 12.21 c
47.049	C2H7O+	ethanol*	0.98 ± 0.52 d	2304.83 ± 165.82 a	1791.74 ± 45.65 b	2790.15 ± 341.90 c	2382.71 ± 159.63 ac
49.012	CH5S+	methanethiol	0.84 ± 0.28 ab	0.68 ± 0.02 a	0.82 ± 0.11 ab	1.24 ± 0.35 ab	1.59 ± 0.46 b
49.03	CH5O2+.	methanediol	0.01 ± 0.01 d	2.79 ± 0.19 a	2.13 ± 0.03 b	3.38 ± 0.31 c	2.85 ± 0.11 a
53.039	C4H5+	Common fragment (propargyl/allyl-type)	2.02 ± 0.08 c	1.21 ± 0.10 ab	1.57 ± 0.19 a	1.11 ± 0.23 b	1.14 ± 0.09 b
55.052	C4H7+	alkene / cycloalkene / terpene / dehydrated alcohol fragment	33.02 ± 6.89 b	3.03 ± 1.08 a	5.85 ± 1.67 a	2.87 ± 2.02 a	2.89 ± 0.93 a
57.035	C3H5O+	acrolein fragment/	7.52 ± 0.51 a	7.16 ± 0.45 a	7.10 ± 0.27 a	10.48 ± 2.04 b	11.24 ± 0.99 b

		acrolein (2-propenal).					
57.07	C4H9+	butanol fragment / dehydrated alcohol fragment	2.94 ± 0.15 c	104.37 ± 7.95 ab	83.99 ± 3.08 a	122.84 ± 17.85 b	118.84 ± 9.64 b
59.049	C3H7O+	acetone*	16.52 ± 1.89 c	242.03 ± 11.04 a	218.31 ± 6.74 a	322.25 ± 36.35 b	338.33 ± 25.07 b
61.029	C2H5O2+	acetic acid*	2.62 ± 0.64 a	26.90 ± 1.81 ab	21.02 ± 11.94 a	80.33 ± 19.01 c	49.96 ± 10.07 b
61.066	C3H9O+	2-propanol*	0.02 ± 0.01 d	2.44 ± 0.25 a	2.65 ± 0.05 a	4.59 ± 0.90 b	5.99 ± 0.65 c
63.044	C2H7O2+	Cluster of acetaldehyde	1.07 ± 0.01 d	51.84 ± 4.78 a	42.31 ± 1.57 ab	31.09 ± 4.47 c	34.47 ± 4.67 bc
69.071	C5H9+	Isoprene/ 3-hexen-2-ol	23.03 ± 1.86 b	4.18 ± 0.09 a	4.92 ± 0.81 a	3.25 ± 0.93 a	3.87 ± 0.41 a
71.014	C3H3O2+	fragment (common from acids/esters)	0.07 ± 0.00 c	0.25 ± 0.01 a	0.21 ± 0.01 a	0.44 ± 0.10 b	0.43 ± 0.05 b
71.05	C4H7O+	2-butenal / methyl vinyl ketone (MVK)	1.71 ± 0.28 a	1.74 ± 0.07 a	1.96 ± 0.08 a	2.40 ± 0.55 a	2.40 ± 0.21 a
71.086	C5H11+	methyl butanol/ pentanol	3.01 ± 0.11 ab	2.17 ± 0.14 a	2.75 ± 0.31 ab	3.61 ± 0.53 bc	4.18 ± 0.51 c
73.067	C4H9O+	butanal*	1.70 ± 0.44 b	6.44 ± 0.25 a	6.20 ± 0.38 a	5.14 ± 1.13 a	6.49 ± 0.54 a
75.048	C3H7O2+	propanoic acid, hydroxyacetone (acetol), and methyl acetate	0.21 ± 0.02 c	0.71 ± 0.04 a	0.52 ± 0.06 a	1.15 ± 0.21 b	1.00 ± 0.07 b
77.023	C2H5O3+	2-Hydroxyacetic acid (glycolic acid)	0.02 ± 0.00 d	4.58 ± 0.63 ab	2.79 ± 0.15 a	7.51 ± 1.93 c	5.23 ± 0.68 bc
77.06	C3H9O2+	Propylene glycol	0.90 ± 0.11 a	0.95 ± 0.14 a	0.83 ± 0.14 a	1.01 ± 0.12 a	0.78 ± 0.11 a
79.053	C6H7+	benzene	0.14 ± 0.00 c	1.40 ± 0.07 a	1.29 ± 0.05 a	1.91 ± 0.29 b	2.05 ± 0.18 b
81.067	C6H9+	Monoterpene fragment	3.92 ± 2.52 a	3.37 ± 0.31 a	5.20 ± 0.98 a	2.79 ± 0.48 a	3.03 ± 0.74 a
85.068	C5H9O+	2-pentenal / 1-penten-3-one	0.91 ± 0.06 b	0.30 ± 0.00 a	0.37 ± 0.02 a	0.31 ± 0.06 a	0.34 ± 0.03 a
85.101	C6H13+	hexanol	0.49 ± 0.04 b	0.22 ± 0.02 a	0.30 ± 0.01 a	0.27 ± 0.06 a	0.27 ± 0.04 a
89.06	C4H9O2+	acetoin /butanoic acid/ isobutyric acid */ ethyl acetate	1.56 ± 0.25 b	0.72 ± 0.16 a	2.16 ± 0.30 b	0.80 ± 0.25 a	0.80 ± 0.22 a
91.056	C4H11S+	butanethiol isomers (1-/2-/iso-/tert-)	0.27 ± 0.12 c	9.34 ± 0.70 a	7.09 ± 1.56 a	17.62 ± 3.45 b	17.11 ± 1.42 b
91.076	C4H11O2+	2,3−butanediol*	1.30 ± 0.44 ab	2.07 ± 0.20 a	1.36 ± 0.16 ab	1.15 ± 0.08 b	1.57 ± 0.40 ab
93.037	C6H5O+	Phenolic / Aromatic fragment	0.00 ± 0.00 c	8.61 ± 1.40 a	5.34 ± 0.13 b	6.83 ± 1.71 ab	6.04 ± 1.15 ab
97.066	C6H9O+	Cyclohexenone / 2,4-Hexadienal	2.19 ± 0.01 ab	2.49 ± 0.12 a	2.69 ± 0.19 a	2.24 ± 0.09 ab	1.73 ± 0.43 b

101.095	C6H13O+	hexanal/ 2-methyl pentanal*	0.11 ± 0.01 b	0.06 ± 0.01 a	0.07 ± 0.01 a	0.07 ± 0.01 a	0.07 ± 0.01 a
103.08	C5H11O2+	2-methyl butanoic acid*	24.99 ± 5.39 b	4.96 ± 0.77 a	7.99 ± 1.01 a	5.10 ± 1.50 a	5.26 ± 0.58 a
105.09	C5H13O2+	1,2-Pentanediol	0.12 ± 0.01 c	0.41 ± 0.02 a	0.30 ± 0.02 a	0.72 ± 0.14 b	0.62 ± 0.04 b
107.049	C7H7O+	benzaldehyde*	0.31 ± 0.06 a	0.45 ± 0.04 ab	0.35 ± 0.01 a	0.77 ± 0.19 c	0.67 ± 0.09 bc
111.044	C6H7O2+	5-methylfurfural / 5-methyl-2-furancarboxaldehyde	5.17 ± 2.97 ab	4.65 ± 0.59 ab	8.54 ± 2.30 a	2.01 ± 0.37 b	2.90 ± 1.42 b
111.113	C8H15+	1-octen-3-ol	0.04 ± 0.01 c	0.12 ± 0.01 a	0.13 ± 0.01 a	0.11 ± 0.01 ab	0.09 ± 0.00 b
113.095	C7H13O+	2-heptenal	17.21 ± 1.36 b	0.71 ± 0.15 a	0.32 ± 0.01 a	0.28 ± 0.06 a	0.34 ± 0.06 a
117.091	C6H13O2+	ethyl isobutyrate or hexanoic acid or ethyl butyrate*	2.46 ± 0.39 b	0.62 ± 0.06 a	0.98 ± 0.25 a	0.54 ± 0.18 a	0.53 ± 0.07 a
121.066	C8H9O+	benzeneacetaldehyde (phenylacetaldehyde)*	0.05 ± 0.00 d	1.72 ± 0.08 ab	1.41 ± 0.20 a	2.13 ± 0.43 b	3.93 ± 0.18 c
133.057	C5H9O4+	Glutaric acid (pentanedioic acid)	0.07 ± 0.02 c	0.33 ± 0.03 a	0.25 ± 0.00 b	0.31 ± 0.04 ab	0.28 ± 0.03 ab
			0.00 ± 0.00 d	0.01 ± 0.00 ab	0.01 ± 0.00 a	0.02 ± 0.00 c	0.02 ± 0.01 bc
139.044	C7H7O3+	hydroxybenzoic acid isomers: salicylic (o-HBA), m-HBA, p-HBA	0.00 ± 0.00 a	0.01 ± 0.00 a	0.01 ± 0.00 a	0.01 ± 0.00 a	0.00 ± 0.00 a
143.11	C8H15O2+	Hexenyl acetate isomers (e.g., 3-hexen-1-yl acetate)	0.09 ± 0.01 b	0.04 ± 0.00 a	0.05 ± 0.01 a	0.04 ± 0.01 a	0.04 ± 0.00 a

Table S7. Tentatively identified mass peaks from the nondestructive PTR–ToF–MS analysis in *Agaricus bisporus* RTS conditions. For each mass peak, the mean ± SD values for each sample are accompanied by Tukey's homogeneous group letters, indicating significant differences ($p < 0.05$) across the samples.

m/z	formula	identification	day1	day8		day15	
			STD	STD	SB	STD	SB
31.019	CH3O+	Unspecific fragment	1.16 ± 0.16	0.04 ± 0.01 a	0.03 ± 0.00 a	0.22 ± 0.04 b	0.04 ± 0.00 a
33.034	CH5O+	methanol	36.19 ± 2.65	0.03 ± 0.00 a	0.04 ± 0.00 b	0.06 ± 0.00 b	0.04 ± 0.00 a
39.022	C3H3+	aromatic / diene / Alkyne fragment	1.13 ± 0.60	0.07 ± 0.03 a	0.12 ± 0.03 a	1.69 ± 0.19 b	0.18 ± 0.11 a

41.039	C3H5+	alkyl fragment	1.42 ± 0.06	0.04 ± 0.03 a	0.11 ± 0.12 a	9.42 ± 3.33 b	1.03 ± 0.98 a
43.018	C2H3O+	acetic acid fragment	12.80 ± 1.01	0.25 ± 0.09 a	0.06 ± 0.04 a	0.38 ± 0.29 a	0.10 ± 0.05 a
43.054	C3H7+	Common fragment	0.70 ± 0.10	0.24 ± 0.01 a	0.23 ± 0.07 a	0.17 ± 0.04 a	0.15 ± 0.04 a
45.033	C2H5O+	Acetaldehyde	21.79 ± 1.63	0.05 ± 0.01 a	0.12 ± 0.00 a	0.62 ± 0.10 a	0.76 ± 0.41 b
47.049	C2H7O+	ethanol*	5.60 ± 4.99	0.13 ± 0.02 a	0.14 ± 0.02 a	0.54 ± 0.16 b	0.20 ± 0.01 a
49.012	CH5S+	methanethiol	0.28 ± 0.06	0.02 ± 0.00 a	0.02 ± 0.00 a	0.08 ± 0.01 b	0.03 ± 0.01 a
49.03	CH5O2+	methanediol	0.03 ± 0.01	0.01 ± 0.00 a	0.01 ± 0.00 a	0.02 ± 0.01 a	0.02 ± 0.01 a
53.042	C4H5+	Common fragment (propargyl/allyl-type)	0.22 ± 0.02	0.01 ± 0.00 a	0.01 ± 0.00 a	0.02 ± 0.00 b	0.01 ± 0.00 a
57.033	C3H5O+	acrolein fragment/ acrolein (2-propenal).	2.31 ± 0.42	0.06 ± 0.01 a	0.11 ± 0.01 b	0.07 ± 0.01 a	0.10 ± 0.02 b
57.07	C4H9+	butanol fragment / dehydrated alcohol fragment	0.79 ± 0.09	1.88 ± 0.89 a	3.81 ± 1.02 b	53.06 ± 4.90 a	14.36 ± 5.42 a
59.049	C3H7O+	acetone*	35.31 ± 2.46	50.08 ± 16.71 a	84.85 ± 9.73 b	130.67 ± 15.70 a	153.55 ± 70.00 a
61.029	C2H5O2+	acetic acid*	11.62 ± 1.61	5.48 ± 2.60 a	25.93 ± 21.78 b	136.68 ± 14.15 a	62.73 ± 25.23 a
61.066	C3H9O+	2-propanol*	0.00 ± 0.00	2.56 ± 1.33 a	6.38 ± 0.26 b	55.52 ± 4.98 a	27.99 ± 11.58 a
63.044	C2H7O2+	Cluster of acetaldehyde	1.38 ± 0.09	19.12 ± 14.10 a	37.93 ± 20.88 b	1289.51 ± 116.65 a	158.61 ± 104.57 a
69.072	C5H9+	Isoprene/ 3-hexen-2-ol	0.27 ± 0.07	1.06 ± 0.75 a	2.51 ± 0.75 b	21.58 ± 1.26 a	16.29 ± 10.94 a
71.014	C3H3O2+	fragment (common from acids/esters)	0.02 ± 0.00	113.76 ± 67.00 a	141.60 ± 86.69 a	1870.41 ± 102.63 a	405.55 ± 528.95 a
71.05	C4H7O+	2-butenal / methyl vinyl ketone (MVK)	0.52 ± 0.12	60.38 ± 80.94 a	134.29 ± 176.35 a	7669.62 ± 1110.53 a	560.30 ± 637.84 a
71.086	C5H11+	methyl butanol/ pentanol	0.20 ± 0.03	0.09 ± 0.02 a	0.13 ± 0.03 a	0.19 ± 0.02 a	0.13 ± 0.07 ab
73.064	C4H9O+	butanal*	0.00 ± 0.00	0.06 ± 0.02 a	0.09 ± 0.05 a	2.47 ± 0.30 b	0.36 ± 0.23 a
75.044	C3H7O2+	propanoic acid, hydroxyacetone (acetol), and methyl acetate	0.72 ± 0.04	0.20 ± 0.02 a	0.22 ± 0.01 a	0.72 ± 0.03 b	0.25 ± 0.06 a
75.08	C4H11O+	1-Butanol / 2-Butanol / Isobutanol	0.01 ± 0.00	2.68 ± 1.03 a	6.97 ± 0.55 b	26.69 ± 2.89 a	24.31 ± 6.52 a

77.023	C2H5O3+	2-Hydroxyacetic acid (glycolic acid)	0.40 ± 0.05	1.16 ± 1.05 a	1.52 ± 0.84 a	127.68 ± 20.49 b	6.66 ± 5.30 a
77.058	C3H9O2+	Propylene glycol	0.87 ± 0.10	119.45 ± 60.24 a	391.48 ± 59.10 b	822.14 ± 198.59 a	1199.18 ± 269.80 a
79.04	C2H7O3+	Acetic acid cluster	1.60 ± 0.30	7.38 ± 1.15 a	9.06 ± 1.40 a	21.07 ± 3.12 b	11.06 ± 3.59 ab
81.033	C5H5O+	Furan fragment	0.03 ± 0.01	0.30 ± 0.22 a	1.07 ± 0.06 a	7.18 ± 0.96 a	5.15 ± 1.93 ab
85.069	C5H9O+	2-Pentenal	0.09 ± 0.00	9.32 ± 8.82 a	12.97 ± 13.01 a	738.59 ± 33.67 a	59.22 ± 79.17 a
85.104	C6H13+	Hexanol fragment / Hexene isomers	0.06 ± 0.01	0.11 ± 0.03 a	0.04 ± 0.04 a	0.32 ± 0.04 b	0.04 ± 0.05 a
89.059	C4H9O2+	Butanone / Butanal	0.25 ± 0.06	0.03 ± 0.01 a	0.06 ± 0.00 a	0.71 ± 0.13 b	0.22 ± 0.06 a
95.086	C7H11+	Monoterpene fragment	0.07 ± 0.01	0.34 ± 0.23 a	0.43 ± 0.33 a	3.79 ± 0.96 b	0.52 ± 0.50 a
97.067	C6H9O+	Cyclohexenone / 2,4-Hexadienal	0.06 ± 0.01	0.12 ± 0.01 a	0.15 ± 0.04 a	1.53 ± 0.47 b	0.40 ± 0.34 a
99.047	C5H7O2+	Furfuryl alcohol / Furan-fragment water cluster	0.08 ± 0.00	0.35 ± 0.61 a	0.39 ± 0.67 a	67.11 ± 9.16 b	0.29 ± 0.50 a
101.097	C6H13O+	Hexanal *	0.06 ± 0.02	0.82 ± 0.20 a	1.23 ± 0.14 b	4.50 ± 0.33 b	1.93 ± 0.70 ab
103.039	C4H7O3+	Butanoic acid, 2-oxo	0.05 ± 0.01	0.04 ± 0.04 a	0.05 ± 0.06 a	34.69 ± 9.27 b	0.44 ± 0.57 a
103.075	C5H11O2+	Pentanoic acid	0.07 ± 0.01	0.77 ± 0.20 a	0.95 ± 0.21 a	11.32 ± 0.88 b	1.97 ± 0.80 a
105.088	C5H13O2+	1,2-Pentanediol/ 2,3-Pentanediol	0.02 ± 0.00	4.43 ± 2.39 a	16.04 ± 2.50 b	31.20 ± 9.31 a	36.76 ± 14.27 a
107.057	C7H7O+	benzaldehyde*	0.06 ± 0.01	1.32 ± 0.15 a	1.50 ± 0.19 a	2.66 ± 0.32 a	1.59 ± 0.39 ab
111.047	C6H7O2+	5-methylfurfural / 5-methyl-2-furancarboxaldehyde	0.14 ± 0.02	0.07 ± 0.01 a	0.07 ± 0.01 a	0.05 ± 0.02 a	0.08 ± 0.01 a
117.090	C6H13O2+	ethyl isobutyrate or hexanoic acid or ethyl butyrate*	0.04 ± 0.00	0.07 ± 0.01 a	0.07 ± 0.02 a	0.55 ± 0.10 b	0.09 ± 0.03 a
121.071	C8H9O+	benzeneacetaldehyde (phenylacetaldehyde)*	0.06 ± 0.01	0.04 ± 0.01 a	0.05 ± 0.00 a	0.03 ± 0.01 a	0.06 ± 0.02 b
131.108	C7H15O2+	Pentyl acetate / Heptanoic acid	0.03 ± 0.00	0.68 ± 0.70 a	1.07 ± 1.19 a	30.18 ± 6.58 b	1.78 ± 1.52 a
133.049	C5H9O4+	Glutaric acid (Pentanedioic acid)	0.01 ± 0.00	0.02 ± 0.01 a	0.03 ± 0.01 a	1.87 ± 0.49 b	0.04 ± 0.03 a
139.046	C7H7O3+	hydroxybenzoic acid isomers: salicylic (o-HBA), m-HBA, p-HBA	0.01 ± 0.00	0.04 ± 0.00 a	0.04 ± 0.00 a	0.10 ± 0.02 a	0.08 ± 0.03 ab
143.11	C8H15O2+	Hexenyl acetate isomers (e.g., 3-	0.08 ± 0.00	0.06 ± 0.00 a	0.05 ± 0.00 a	0.11 ± 0.01 b	0.07 ± 0.00 a

		hexen-1-yl acetate)					

Table S8. Tentatively identified mass peaks from the nondestructive PTR–ToF–MS analysis in *Agaricus bisporus* during refrigerated storage (4 °C). For each mass peak, the mean ± SD values for each sample are accompanied by Tukey's homogeneous group letters, indicating significant differences ($p < 0.05$) across the samples.

m/z	formula	identification	day1	day8		day15	
			STD	STD	SB	STD	SB
31.019	CH3O+	Unspecific fragment	1.16 ± 0.16	0.04 ± 0.00 a	0.04 ± 0.00 a	0.05 ± 0.02 a	0.03 ± 0.00 a
33.034	CH5O+	methanol	36.19 ± 2.65	0.04 ± 0.00 a	0.04 ± 0.00 a	0.04 ± 0.01 a	0.04 ± 0.00 a
39.022	C3H3+	aromatic / diene / Alkyne fragment	1.13 ± 0.60	0.07 ± 0.01 a	0.10 ± 0.01 a	0.42 ± 0.35 a	0.12 ± 0.07 a
41.039	C3H5+	alkyl fragment	1.42 ± 0.06	0.02 ± 0.00 a	0.05 ± 0.03 a	1.42 ± 1.22 a	0.31 ± 0.32 a
43.018	C2H3O+	acetic acid fragment	12.80 ± 1.01	0.10 ± 0.05 a	0.09 ± 0.03 a	0.18 ± 0.16 a	0.10 ± 0.00 a
43.054	C3H7+	Common fragment	0.70 ± 0.10	0.17 ± 0.01 a	0.18 ± 0.01 a	0.10 ± 0.02 a	0.15 ± 0.02 a
45.033	C2H5O+	Acetaldehyde	21.79 ± 1.63	0.05 ± 0.01 a	0.07 ± 0.01 a	0.13 ± 0.09 a	0.28 ± 0.06 a
47.049	C2H7O+	ethanol*	5.60 ± 4.99	0.16 ± 0.02 a	0.17 ± 0.04 a	0.18 ± 0.11 a	0.17 ± 0.04 a
49.012	CH5S+	methanethiol	0.28 ± 0.06	0.02 ± 0.00 a	0.02 ± 0.00 a	0.03 ± 0.01 a	0.03 ± 0.00 a
49.03	CH5O2+	methanediol	0.03 ± 0.01	0.01 ± 0.00 a	0.01 ± 0.00 a	0.01 ± 0.00 a	0.02 ± 0.00 b
53.042	C4H5+	Common fragment (propargyl/allyl-type)	0.22 ± 0.02	0.01 ± 0.00 a	0.01 ± 0.00 a	0.01 ± 0.00 a	0.01 ± 0.00 a
57.033	C3H5O+	acrolein fragment/ acrolein (2-propenal).	2.31 ± 0.42	0.06 ± 0.01 a	0.11 ± 0.03 b	0.04 ± 0.01 a	0.08 ± 0.01 b
57.07	C4H9+	butanol fragment / dehydrated alcohol fragment	0.79 ± 0.09	1.46 ± 0.27 a	2.72 ± 0.54 b	20.54 ± 16.98 a	8.25 ± 2.75 a
59.049	C3H7O+	acetone*	35.31 ± 2.46	49.07 ± 10.55 a	81.45 ± 9.65 b	95.21 ± 75.31 a	131.72 ± 25.50 a
61.029	C2H5O2+	acetic acid*	11.62 ± 1.61	20.03 ± 25.60 a	8.82 ± 0.65 a	34.33 ± 30.95 a	46.50 ± 4.44 a
61.066	C3H9O+	2-propanol*	0.00 ± 0.00	2.28 ± 0.57 a	4.33 ± 0.30 b	15.06 ± 13.17 a	17.78 ± 5.46 a
63.044	C2H7O2+	Cluster of acetaldehyde	1.38 ± 0.09	12.89 ± 1.96 a	27.98 ± 10.68 b	402.68 ± 353.70 a	76.67 ± 41.69 a
69.072	C5H9+	Isoprene/ 3-hexen-2-ol	0.27 ± 0.07	0.85 ± 0.24 a	1.85 ± 0.44 b	7.11 ± 5.65 a	11.11 ± 5.27 a

71.014	C3H3O2+	fragment (common from acids/esters)	0.02 ± 0.00	90.80 ± 10.26 a	141.32 ± 49.91 a	1611.68 ± 1456.56 a	127.83 ± 144.55 a
71.05	C4H7O+	2-butenal / methyl vinyl ketone (MVK)	0.52 ± 0.12	11.62 ± 0.71 a	98.77 ± 100.96 a	2188.75 ± 1928.85 a	251.06 ± 268.71 a
71.086	C5H11+	methyl butanol/ pentanol	0.20 ± 0.03	0.09 ± 0.06 a	0.09 ± 0.03 a	0.04 ± 0.02 a	0.07 ± 0.02 a
73.064	C4H9O+	butanal*	0.00 ± 0.00	0.05 ± 0.01 a	0.08 ± 0.02 a	0.85 ± 0.71 a	0.19 ± 0.09 a
75.044	C3H7O2+	propanoic acid, hydroxyacetone (acetol), and methyl acetate	0.72 ± 0.04	0.19 ± 0.02 a	0.23 ± 0.02 a	0.30 ± 0.15 a	0.23 ± 0.03 a
75.08	C4H11O+	1-Butanol / 2-Butanol / Isobutanol	0.01 ± 0.00	2.66 ± 0.56 a	4.32 ± 0.87 b	7.87 ± 6.23 a	15.63 ± 2.42 a
77.023	C2H5O3+	2-Hydroxyacetic acid (glycolic acid)	0.40 ± 0.05	0.62 ± 0.08 a	1.70 ± 0.47 b	25.45 ± 26.24 a	3.53 ± 2.57 a
77.058	C3H9O2+	Propylene glycol	0.87 ± 0.10	121.63 ± 41.02 a	221.57 ± 50.22 b	280.20 ± 235.80 a	797.18 ± 87.35 a
79.04	C2H7O3+	Acetic acid cluster	1.60 ± 0.30	7.59 ± 0.21 a	8.78 ± 0.51 a	10.61 ± 4.13 a	6.86 ± 2.88 a
81.033	C5H5O+	Furan fragment	0.03 ± 0.01	0.29 ± 0.12 a	0.63 ± 0.05 a	1.88 ± 1.65 a	3.19 ± 0.92 a
85.069	C5H9O+	2-Pentenal	0.09 ± 0.00	5.05 ± 0.31 a	11.71 ± 8.00 a	238.66 ± 217.35 a	17.21 ± 20.22 a
85.104	C6H13+	Hexanol fragment / Hexene isomers	0.06 ± 0.01	0.09 ± 0.02 a	0.09 ± 0.02 a	0.07 ± 0.07 a	0.00 ± 0.00 a
89.059	C4H9O2+	Butanone / Butanal	0.25 ± 0.06	0.03 ± 0.01 a	0.05 ± 0.01 a	0.15 ± 0.12 a	0.13 ± 0.03 a
95.086	C7H11+	Monoterpene fragment	0.07 ± 0.01	0.27 ± 0.08 a	0.39 ± 0.08 a	1.54 ± 1.65 a	0.39 ± 0.24 a
97.067	C6H9O+	Cyclohexenone / 2,4-Hexadienal	0.06 ± 0.01	0.10 ± 0.02 a	0.19 ± 0.04 a	0.27 ± 0.23 a	0.18 ± 0.02 a
99.047	C5H7O2+	Furfuryl alcohol / Furan-fragment water cluster	0.08 ± 0.00	0.00 ± 0.00 a	0.42 ± 0.73 a	8.73 ± 9.36 b	0.00 ± 0.00 a
101.097	C6H13O+	Hexanal *	0.06 ± 0.02	0.76 ± 0.06 a	1.21 ± 0.34 b	1.98 ± 1.18 a	1.50 ± 0.45 a
103.039	C4H7O3+	Butanoic acid, 2-oxo	0.05 ± 0.01	0.02 ± 0.00 a	0.04 ± 0.03 a	5.18 ± 4.82 b	0.11 ± 0.12 a
103.075	C5H11O2+	Pentanoic acid	0.07 ± 0.01	0.62 ± 0.10 a	0.75 ± 0.13 a	3.92 ± 3.01 a	1.39 ± 0.43 a
105.088	C5H13O2+	1,2-Pentanediol/ 2,3-Pentanediol	0.02 ± 0.00	4.44 ± 1.57 a	8.37 ± 2.13 b	9.63 ± 8.19 a	31.49 ± 4.78 b
107.057	C7H7O+	benzaldehyde*	0.06 ± 0.01	1.33 ± 0.03 a	1.47 ± 0.08 a	1.41 ± 0.63 a	1.14 ± 0.42 a
111.047	C6H7O2+	5-methylfurfural / 5-methyl-2-	0.14 ± 0.02	0.06 ± 0.01 a	0.06 ± 0.00 a	0.06 ± 0.02 a	0.07 ± 0.01 a

		furancarboxaldehyde					
117.090	C6H13O2+	ethyl isobutyrate or hexanoic acid or ethyl butyrate*	0.04 ± 0.00	0.06 ± 0.00 a	0.08 ± 0.01 a	0.17 ± 0.10 a	0.07 ± 0.03 a
121.071	C8H9O+	benzeneacetaldehyde (phenylacetaldehyde)*	0.06 ± 0.01	0.04 ± 0.01 a	0.05 ± 0.01 a	0.03 ± 0.01 a	0.07 ± 0.01 b
131.108	C7H15O2+	Pentyl acetate / Heptanoic acid	0.03 ± 0.00	0.51 ± 0.25 a	0.82 ± 0.29 a	7.55 ± 7.80 a	0.92 ± 0.80 a
133.049	C5H9O4+	Glutaric acid (Pentanedioic acid)	0.01 ± 0.00	0.04 ± 0.01 a	0.04 ± 0.01 a	0.30 ± 0.26 a	0.03 ± 0.01 a
139.046	C7H7O3+	hydroxybenzoic acid isomers: salicylic (o-HBA), m-HBA, p-HBA	0.01 ± 0.00	0.04 ± 0.00 a	0.05 ± 0.00 a	0.04 ± 0.01 a	0.05 ± 0.01 a
143.11	C8H15O2+	Hexenyl acetate isomers (e.g., 3-hexen-1-yl acetate)	0.08 ± 0.00	0.06 ± 0.00 a	0.06 ± 0.00 a	0.07 ± 0.02 a	0.06 ± 0.01 a

Table S9. Tentatively identified mass peaks from the nondestructive PTR–ToF–MS analysis in *Agaricus bisporus* during Ambient temperature (20 °C) storage. For each mass peak, the mean ± SD values for each sample are accompanied by Tukey's homogeneous group letters, indicating significant differences ($p < 0.05$) across the samples.

m/z	formula	identification	day1	day8	
			STD	STD	SB
31.019	CH3O+	Unspecific fragment	1.16 ± 0.16	0.54 ± 0.16 a	0.59 ± 0.17 a
33.034	CH5O+	methanol	36.19 ± 2.65	0.08 ± 0.00 b	0.06 ± 0.00 a
39.022	C3H3+	aromatic / diene / Alkyne fragment	1.13 ± 0.60	6.48 ± 1.61 a	5.57 ± 1.44 a
41.039	C3H5+	alkyl fragment	1.42 ± 0.06	55.62 ± 21.18 a	48.90 ± 14.14 a
43.018	C2H3O+	acetic acid fragment	12.80 ± 1.01	1.46 ± 0.71 a	0.97 ± 0.37 a
43.054	C3H7+	Common fragment	0.70 ± 0.10	0.14 ± 0.01 a	0.15 ± 0.02 a
45.033	C2H5O+	Acetaldehyde	21.79 ± 1.63	4.01 ± 1.24 a	3.64 ± 0.93 a
47.049	C2H7O+	ethanol*	5.60 ± 4.99	0.63 ± 0.08 b	0.48 ± 0.06 a
49.012	CH5S+	methanethiol	0.28 ± 0.06	0.16 ± 0.06 a	0.14 ± 0.03 a
49.03	CH5O2+	methanediol	0.03 ± 0.01	0.01 ± 0.00 a	0.01 ± 0.00 a
53.042	C4H5+	Common fragment (propargyl/allyl-type)	0.22 ± 0.02	0.02 ± 0.00 b	0.02 ± 0.00 a

57.033	C3H5O+	acrolein fragment/ acrolein (2-propenal).	2.31 ± 0.42	0.08 ± 0.00 a	0.10 ± 0.01 a
57.07	C4H9+	butanol fragment / dehydrated alcohol fragment	0.79 ± 0.09	46.20 ± 10.97 a	46.00 ± 2.76 a
59.049	C3H7O+	acetone*	35.31 ± 2.46	72.25 ± 15.72 a	103.17 ± 7.61 b
61.029	C2H5O2+	acetic acid*	11.62 ± 1.61	326.68 ± 73.01 a	380.79 ± 43.08 a
61.066	C3H9O+	2-propanol*	0.00 ± 0.00	113.52 ± 22.11 a	142.18 ± 14.43 a
63.044	C2H7O2+	Cluster of acetaldehyde	1.38 ± 0.09	1222.13 ± 239.99 a	1189.11 ± 203.54 a
69.072	C5H9+	Isoprene/ 3-hexen-2-ol	0.27 ± 0.07	80.54 ± 8.66 a	105.74 ± 14.95 a
71.014	C3H3O2+	fragment (common from acids/esters)	0.02 ± 0.00	3676.00 ± 1009.84 a	3432.70 ± 2045.51 a
71.05	C4H7O+	2-butenal / methyl vinyl ketone (MVK)	0.52 ± 0.12	10626.63 ± 2315.28 a	8051.15 ± 1748.48 a
71.086	C5H11+	methyl butanol/ pentanol	0.20 ± 0.03	1.55 ± 0.75 a	9.06 ± 4.85 b
73.064	C4H9O+	butanal*	0.00 ± 0.00	2.19 ± 0.40 b	1.48 ± 0.28 a
75.044	C3H7O2+	propanoic acid, hydroxyacetone (acetol), and methyl acetate	0.72 ± 0.04	0.67 ± 0.12 b	0.56 ± 0.07 a
75.08	C4H11O+	1-Butanol / 2-Butanol / Isobutanol	0.01 ± 0.00	52.48 ± 11.58 a	61.43 ± 7.25 a
77.023	C2H5O3+	2-Hydroxyacetic acid (glycolic acid)	0.40 ± 0.05	194.40 ± 57.50 a	184.63 ± 24.35 a
77.058	C3H9O2+	Propylene glycol	0.87 ± 0.10	1583.28 ± 527.39 a	1970.50 ± 264.97 a
79.04	C2H7O3+	Acetic acid cluster	1.60 ± 0.30	27.72 ± 18.96 a	27.30 ± 9.79 a
81.033	C5H5O+	Furan fragment	0.03 ± 0.01	19.93 ± 20.30 a	28.73 ± 18.60 a

85.069	C5H9O+	2-Pentenal	0.09 ± 0.00	598.00 ± 129.22 a	722.76 ± 223.92 a
85.104	C6H13+	Hexanol fragment / Hexene isomers	0.06 ± 0.01	1.75 ± 1.07 a	1.84 ± 0.67 a
89.059	C4H9O2+	Butanone / Butanal	0.25 ± 0.06	2.10 ± 0.53 a	1.73 ± 0.71 a
95.086	C7H11+	Monoterpene fragment	0.07 ± 0.01	15.35 ± 6.30 b	6.06 ± 2.53 a
97.067	C6H9O+	Cyclohexenone / 2,4-Hexadienal	0.06 ± 0.01	5.47 ± 2.65 a	3.97 ± 1.71 a
99.047	C5H7O2+	Furfuryl alcohol / Furan-fragment water cluster	0.08 ± 0.00	76.45 ± 26.11 a	57.12 ± 25.24 a
101.097	C6H13O+	Hexanal *	0.06 ± 0.02	8.04 ± 1.37 a	8.82 ± 1.03 a
103.039	C4H7O3+	Butanoic acid, 2-oxo	0.05 ± 0.01	96.51 ± 26.34 b	54.28 ± 16.83 a
103.075	C5H11O2+	Pentanoic acid	0.07 ± 0.01	12.42 ± 2.13 a	12.85 ± 2.15 a
105.088	C5H13O2+	1,2-Pentanediol/ 2,3-Pentanediol	0.02 ± 0.00	49.73 ± 13.25 a	35.92 ± 2.46 a
107.057	C7H7O+	benzaldehyde *	0.06 ± 0.01	5.01 ± 0.96 a	4.63 ± 0.77 a
111.047	C6H7O2+	5-methylfurfural / 5-methyl-2-furancarboxaldehyde	0.14 ± 0.02	0.10 ± 0.05 a	0.08 ± 0.05 a
117.090	C6H13O2+	ethyl isobutyrate or hexanoic acid or ethyl butyrate*	0.04 ± 0.00	1.33 ± 0.29 a	1.12 ± 0.17 a
121.071	C8H9O+	benzeneacetaldehyde (phenylacetaldehyde)*	0.06 ± 0.01	0.04 ± 0.02 a	0.09 ± 0.02 b
131.108	C7H15O2+	Pentyl acetate / Heptanoic acid	0.03 ± 0.00	96.48 ± 34.13 a	60.77 ± 17.25 a
133.049	C5H9O4+	Glutaric acid (Pentanedioic acid)	0.01 ± 0.00	5.38 ± 1.30 b	2.98 ± 0.98 a
139.046	C7H7O3+	hydroxybenzoic acid isomers: salicylic (o-HBA), m-HBA, p-HBA	0.01 ± 0.00	0.36 ± 0.11 a	0.43 ± 0.09 a

143.11	$C_8H_{15}O_2^+$	Hexenyl acetate isomers (e.g., 3-hexen-1-yl acetate)	0.08 ± 0.00	0.17 ± 0.03 a	0.16 ± 0.02 a

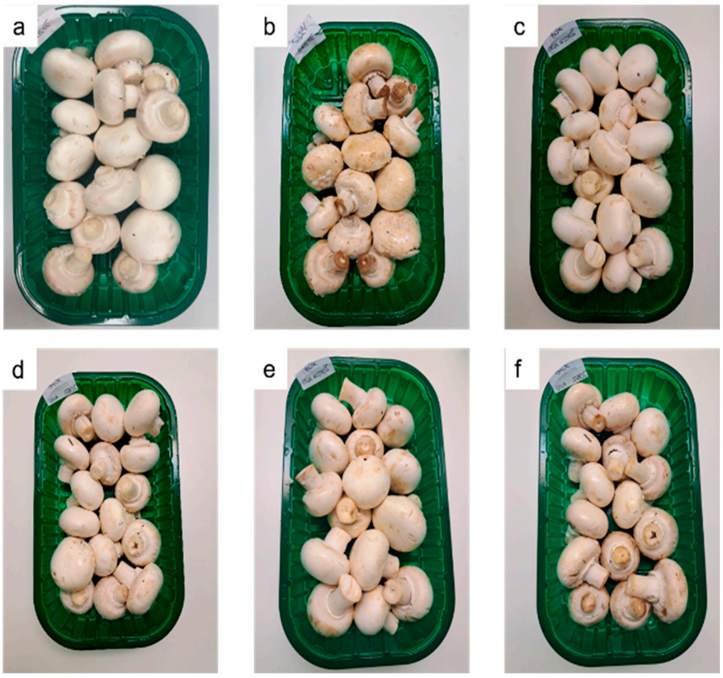

Figure S1. Representative photographs of *Agaricus bisporus* stored in SB packaging. (a) sample at day 0; (b–d) samples after 8 days of storage under ambient, refrigerated, and RTS conditions, respectively; (e,f) samples after 15 days of storage under refrigerated and RTS conditions, respectively.

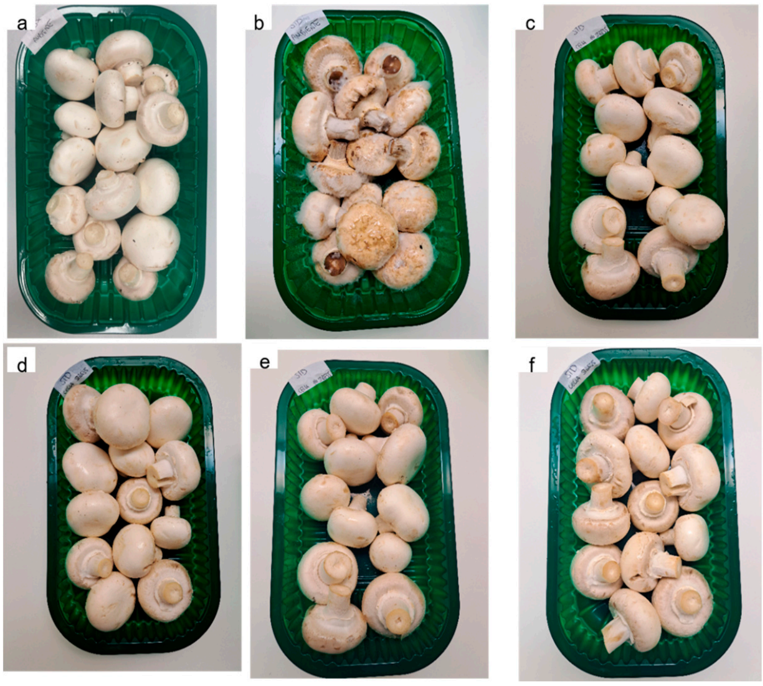

Figure S2. Representative photographs of *Agaricus bisporus* stored in STD packaging. (a) sample at day 0; (b–d) samples after 8 days of storage under ambient, refrigerated, and RTS conditions, respectively; (e,f) samples after 15 days of storage under refrigerated and RTS conditions, respectively.
